# BloodImage: Benchmarking vision transformers for blast detection in digital blood films using public and clinical datasets

**DOI:** 10.1016/j.jpi.2025.100525

**Published:** 2025-10-31

**Authors:** Concetta Piazzese, Sophie Williams, Gregory Slabaugh, Timothy Farren, Tanya Freeman, Laura Aiken, Juswal Dadhra, Stefan Browne, Simon Deltadahl, BloodCounts! Consortium, Suthesh Sivapalaratnam

**Affiliations:** a Barts Life Sciences, Barts Health NHS Trust, London, United Kingdom; b PHURI, Queen Mary University of London, London, United Kingdom; c DERI, Queen Mary University of London, London, United Kingdom; d NHS East and South East London (ESEL) Pathology Partnership, London, United Kingdom; e Clinical Haematology, Barts Health NHS Trust, London, United Kingdom; f Automation Consultants, Reading, United Kingdom; g Department of Applied Mathematics and Theoretical Physics, University of Cambridge, Cambridge, UK; h Blizard, Queen Mary University of London, London, United Kingdom

**Keywords:** Vision transformer (ViT), Data augmentation, Blood films, Leukemia, Blast cells

## Abstract

**Background and objectives:**

Leukemia is one of the most common cancers in the UK and it is usually initially diagnosed through the time-consuming and subjective analysis of blood films by an expert hematologist. When a small number of blast cells may be present on a blood film, it is difficult to detect them even after a thorough review. Automating blood film image analysis could significantly speed up the process and improve diagnostic accuracy. This study benchmarks a machine learning framework based on vision transformers (ViTs) for automated blast detection in digitized blood films, evaluating their generalizability across public and clinical datasets.

**Methods:**

We investigated different training strategies (hold-out/k-fold cross-validation), optimization (Adam or stochastic gradient descent (SGD)), and data preprocessing techniques (data augmentation, Gaussian pyramid downsampling) to assess their impact on the ViT performance when tested using both public (ALL-IDB) and clinical datasets from Barts Health NHS Trust.

**Results:**

Models trained with Adam performed better than those trained with SGD. The best-performing model, ViT2-Adam, achieved the highest accuracy (≥0.86) and area under the receiver operating characteristic curvearea under the curve (AUROC ≥ 0.95), which exceeded other stochastic models demonstrating its potential for integration into clinical diagnostic workflows.

**Conclusions:**

Our findings support the viability of ViTs for clinical integration in blood film analysis. Augmentation, advanced data splitting, and Gaussian downsampling enhance model generalization, offering a promising strategy for resource-limited or high-throughput diagnostic environments.

## Highlights

• Vision transformers benchmarked for blast detection in blood film images.
• Extensive internal and external validation using public and in-house datasets.
• Achieved high accuracy, sensitivity, and specificity across diverse testing scenarios.
• Potential for clinical adoption in hematological diagnostic settings.

## 1 Introduction

Leukemia is the 12th most common cancer in the UK with around 10 302 new leukemia cases every year.^6^ It originates in bone marrow, and results from uncontrolled growth of white blood cells. In leukemia, the abnormal immature white blood cells are not able to fight infection, and they may also impair the ability of the bone marrow to produce red blood cells and platelets leading to fatigue and bleeding.^16^

Early diagnosis and treatment of leukemia is crucial to reduce morbidity and mortality.^8^^,^^12^

The current gold-standard approach for the initial diagnosis of acute leukemia and other hematological disorders involves the manual morphological analysis of blood films, drawing heavily on the expertise and experience of trained hematologists and biomedical scientists. This approach enables a detailed visual assessment of the cells or immature cells such as blasts present in the blood sample at the time of collection, leveraging human morphological expertise to identify subtle abnormalities.^13^ Peripheral blood is spread on a glass slide and treated with a Romanowsky stain, allowing the experienced biomedical scientist or hematologist to examine it under a microscope and to confirm the presence of blasts and type of leukemia. The review process is time-intensive, relies on subjective interpretation, and demands extensive training to cultivate the knowledge and skills necessary for accurate and precise morphological assessment.

We believe automation can improve early diagnosis in hematological conditions. Automation of data analysis and the use of machine learning has applications in healthcare, including diagnostic support and classification of leukemia patients. A decision-support system was proposed by Moshavash et al.^19^ to detect non-overlapping lymphoblast cells in pathology images with an accuracy of 89.81%. Mishra et al.^18^ used a random forest classifier to identify leukemia cells in digitized blood films. The method achieved an accuracy of 99% but it strongly relies on the segmentation performed before classifying the cells. Convolutional neural networks (CNNs) were proposed by Abas et al.^1^ and Atteia et al.^4^ as a diagnostic tool for leukemia.

Recently, transformer-based models^10^^,^^11^^,^^17^^,^^34^ such as vision transformers (ViTs) and shifted window (swin) transformers have emerged as a competitive alternative to CNNs that have traditionally provided state-of-the-art performance in computer vision.

In this work, we benchmark a machine learning framework based on ViT to automatically analyze and identify blasts from blood films. By using public and in-house datasets, we applied a range of training strategies, optimization, and data preprocessing techniques, to perform extensive internal and external validation and compared the performances of different ViT models. This systematic evaluation was designed to assess the robustness and generalizability of each model across varying experimental scenarios and to assess the model's generalizability and clinical applicability across different datasets. The framework is designed to operate with minimal hardware requirements, making it accessible for deployment in resource-limited clinical settings.

## 2 Material and methods

### 2.1 Dataset

**Fig. 1:**
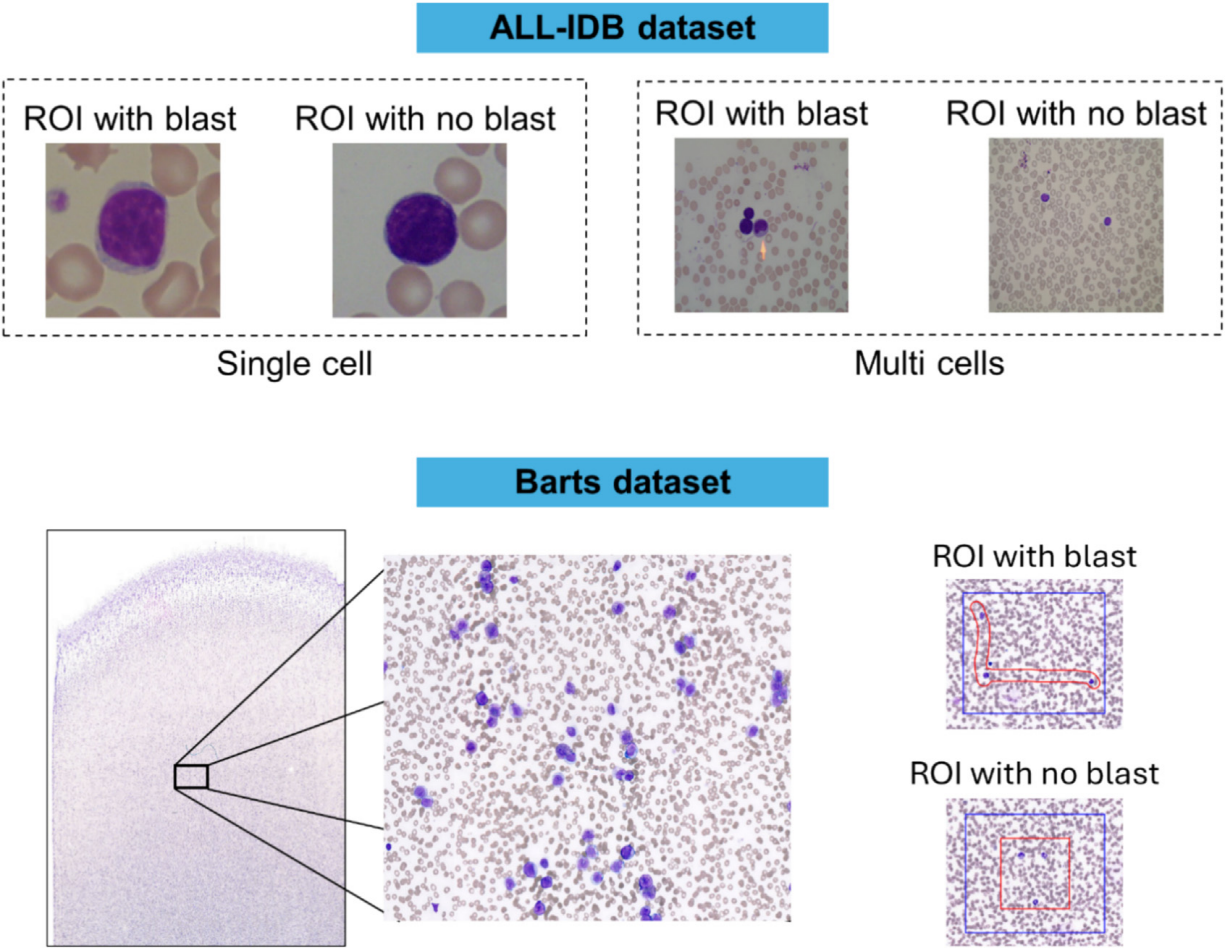
Images from the datasets used in this study. The ALL-IDB contains images of single cells (top left) and multiple cells (top right). The Barts dataset includes digitalized full blood film slides (bottom left). Annotations (bottom right, red contours) provided by two hematologists were transformed to uniform shapes (bottom right, blue contour) to ensure consistency before being extracted and saved as individual images. (For interpretation of the references to color in this figure legend, the reader is referred to the web version of this article.)

In this study, we collected a total of 102 blood film slides (Fig. 1 bottom left) from the Pathology Department at Royal London Hospital, Barts Health NHS Trust, containing a variety of disorders including acute leukemia, chronic leukemia, and non-malignant hematological conditions as well as slides from healthy (normal) patients. Slide preparation was done using the integrated Sysmex SP-50 slidemaker/stainer and were processed using a standalone 3D Histech Pannoramic Midi II Scanner System and a standalone 3D Histech Pannoramic 1000 Scanner System. Two expert hematologists annotated the digitized slides using QuPath^5^ to identify regions of interest (ROIs) containing blasts and regions without blasts. Given the variability in size and shape of the outlined ROIs, they were standardized to uniform shapes (Fig. 1 bottom right) to ensure consistency before being extracted and saved as individual images. This process resulted in a total of 289 individual images derived from the original 102 blood film slides. Although each slide corresponds to a unique patient, multiple ROIs were extracted from some slides, accounting for the higher number of individual images. Each image was categorized based on the presence or absence of blasts.

We also included in our study the microscopic blood cell images from the public ALL-IDB dataset.^9^ It includes images with blasts (immature blood cells) and regions without blasts from patients with acute lymphoblastic leukemia (ALL) and healthy individuals. The ALL-IDB dataset is considered highly reliable because expert oncologists provided detailed classifications and positions of ALL lymphoblasts for each image. The dataset is divided into two subsets: ALL-IDB1, containing 108 images of single cells, and ALL-IDB2, containing 260 images with multiple cells (Fig. 1 top).

Table 1 shows the number of samples for each subtype within each dataset.

**Table 1:** Datasets used in the study, detailing the image type and the total number of images in each dataset.

Dataset	Healthy	Pathological	Image type	Total images
ALL-IDB1	59	49	Multi cells	108
ALL-IDB2	130	130	Single cell	260
Barts	144	145	Multi cells	289

### 2.2 Data augmentation

Data augmentation was applied to improve the diversity and robustness of the training dataset, and ultimately improving the model's performance. The augmentation process was applied only to the original training images, with each transformation applied one at a time before starting the training process, resulting in an 8-fold increase in the dataset size. The transformations included in the data augmentation process were:

• Random vertical flip: Each image in the dataset gets flipped vertically ensuring that the model sees images that are also mirrored vertically from the original, so that it can be invariant to changes associated with vertical orientation.
• Random horizontal flip: The same treatment of flipping the images horizontally to include horizontal mirroring and make the model better at identification independent of the left–right orientation of objects.
• Random rotation: These images were randomly rotated within ±40 degrees. This transformation simulates scenarios where the objects might appear at a different angle, which helps make the model more robust to changes in orientation.
• Random shear: A random affine shear transformation of up to 20 degrees was applied. This transformation distorts images along one axis; as such, it simulates changes in perspective or camera angles that allow the model to generalize from a variety of viewpoints.
• Random horizontal translation: Images are horizontally randomly translated to at most 40% of the image width. This affine translation introduces lateral shifts so that the model is trained on scenarios when an object is not at the center of the image.
• Random vertical translation: The images were vertically translated by up to 40% of the image height. This vertical shift makes the model sensitive to changes in the vertical position of the objects in the images.
• Random zoom and resizing: Images were zoomed between 10% and 30%, and then resized to 1024 × 1024 pixels. This procedure introduced some disparity in object scales across the image, which increased the model's perception of objects at various scales. The overall sharpness or resolution of the picture stayed the same after resizing.
• Gaussian pyramid downsampling: Two stages of Gaussian pyramid downsampling were done to further increase the dataset. This is a way by which the image is taken through a progressive decrease in resolution and then it is resized to the target size. Unlike the previously mentioned techniques, such as random zoom and resizing where the sharpness and focus are manipulated, using Gaussian Pyramid Downsampling modifies the resolution of the image (as it might happen with blood images digitized using different scanners) and introduces blurriness or variable focus.

These transformations were carefully selected from previous published papers^2^^,^^21^^,^^29^ to introduce realistic variations that the model might see when deployed in clinical settings.

### 2.3 VIT architecture

The basic ViT architecture^10^ is used in this study to process colored image data by breaking down each image into smaller, and hence more manageable components. The process starts by dividing the input images into square patches, which are then flattened and combined into a sequential format suitable for transformer processing. To maintain the spatial relationships between these patches, positional embeddings are added, so that the model retains information about the original positions of the patches within the image. The core architecture of ViT consists of multiple self-attention mechanisms, each one aiming at identifying relationships between the image patches and capturing various features across the entire image. The transformer then processes the patches through a series of blocks combining attention layers with feed-forward neural networks. Each block refines the image representation by repeatedly applying attention and transformation, thereby enhancing the model's ability to extract meaningful patterns.

Given the limited size and complexity of our dataset, we employed a ViT architecture that closely resembles a ViT-B/16 variant, with 8 transformer layers and 4 attention heads per block, each using a wider 1024-dimensional hidden space. This shallow-but-wide configuration was designed to balance model capacity with the risk of overfitting, while maintaining a competitive parameter count (∼86 M) by reducing depth and attention complexity. The ViT configuration used in this study is provided in Table 2.

**Table 2:** Configurations of ViT model used in the study. The model processes images by dividing them into 16 patches and passing them through 8 layers (transformer blocks). Each layer includes four attention heads per block, capturing spatial dependencies within the image. The hidden dimensionality (width) is set to 1024, allowing the model to effectively capture and process intricate details within the image data. The multilayer perceptron (MLP) ratio is 4, indicating that the MLP block expands the hidden dimension by a factor of 4 before reducing it back to the original size. The model outputs predictions for 2 classes, with a total of 86 M parameters.

Dataset	Image type
Image patches	16
Layers	8
Attention heads	4 (per block)
Width (Hidden dimensionality)	1024
MLP ratio	4
Output classes	2
Parameters	86 085 634

### 2.4 Optimization techniques

We used two different optimization algorithms, Adam and Stochastic Gradient Descent (SGD), to train the models. Adam^15^^,^^24^ is a gradient-based optimization algorithm that combines first-moment estimation (momentum) and second-moment estimation (adaptive per-parameter scaling) to adjust the learning rate for each parameter dynamically. It also applies bias correction to compensate for the initialization bias in the moment estimates, which helps stabilize early training. These properties typically allow Adam to achieve faster and more stable convergence with minimal hyperparameter tuning. SGD^26^ is a simple optimization approach that iteratively updates the model weights based on the loss function, making it effective for large-scale machine learning tasks. Both optimizers were used with a learning rate of 3 × 10^−7^, which is a dimensionless hyperparameter controlling the step size during training. We explored both optimizers in the context of our problem to determine which provided better performance.

### 2.5 Training, validation, and testing procedure

We trained the models over several epochs, with a maximum of 100 epochs, with performance evaluated at the end of each epoch using a validation dataset. Input images were automatically resized during the training phase to 1024 × 1024 pixels. Key metrics, such as loss and accuracy, were tracked to monitor model performance. For the loss function, we used cross-entropy loss as the criterion to compute the discrepancy between the predicted and the target labels. To prevent overfitting, early stopping was implemented to stop the training if any of the following occurred: the validation loss did not improve after 10 consecutive epochs or divergence between training and validation loss for 5 consecutive epochs. Data augmentation was applied to the training set only.

The model parameters corresponding to the epoch with the best validation performance were saved for further analysis. This approach ensures that the most effective version of the model is preserved, even if early stopping is triggered.

### 2.6 Experiments

We conducted a series of experiments systematically organized into five configurations, labeled ViT0 through VIT4, to explore the effects of various training strategies, optimization, and data preprocessing techniques on model performance. The experiments gradually increase in complexity, from a basic model with no augmentation (ViT0) to a highly augmented model with advanced image processing techniques like Gaussian pyramids applied to both original and augmented data (ViT4). All models (VIT0–VIT4) were trained and validated on an Ubuntu 22.04 machine with an Intel® Xeon® E5-2690 v4 CPU @ 2.60GHz and a Nvidia Tesla V100-PCIE-16GB GPU.

We performed internal and external testing of the models by using the available datasets. For the internal testing, the combined dataset ALL-IDB1 + ALL-IDB2 + Barts was used for training/validation/testing. The splitting applied in this case was either 60:20:20 for training, validation, and testing when hold-out data splitting was used or 80:20 for training/validation and testing followed by a 4-fold splitting for the training–validation set for more advanced data splitting. After k-fold cross-validation, the model is retrained on the entire training/validation set (all k folds combined) and evaluated on the test set to obtain the final performance metrics. For all experiments, the training data size, augmented training data size, and validation data size varied based on the augmentation and k-fold strategies, with different fold sizes leading to slight variations in validation sets.

For the external testing, each possible ViT configuration was trained/validated on one dataset or combination of datasets and tested on the remaining one/ones. For instance, a ViT model trained/validated on the ALL-IDB1 dataset, was tested on the ALL-IDB2 dataset and vice versa, while when the model was trained/validated on the combined dataset ALL-IDB1 + ALL-IDB2, it was then tested on the Barts dataset. The training/validation splitting for external testing was either 80:20 or 4-fold cross-validation.

Wherever possible, data splits were performed at the patient level to prevent data leakage. For the Barts Health dataset, patient identifiers were available, allowing all ROIs from the same patient to be included exclusively in either the training, validation, or test sets. For the ALL-IDB datasets, such information was not available, and each image was therefore assumed to originate from a different patient, following common practice in prior studies.

More detailed information about all the experiments performed in this study and the training, validation, and testing strategies used for the internal and external validation is provided in Table 3, Table 4.

**Table 3:** Configurations and dataset splits for internal validation experiments. The experiments include ViT models with no augmentation, with augmentation, and with k-fold cross-validation (4 folds). Additionally, Gaussian pyramid transformations are applied in some cases. The dataset used is a combination of ALL-IDB1, ALL-IDB2, and Barts. The split ratios and dataset sizes for training, validation, and testing are provided for each configuration. The asterisk (*) in the tables indicates that one fold in the 4-fold cross-validation resulted in different training and validation sizes compared to the other folds.

Label	Description	Optimization algorithm	Training/Validation split ratio	Training/Validation dataset	Total dataset size	Training set size	Augmented training set size	Validation set size	Testing set size
ViT0	No augmentation No k-fold cross-validation	Adam/SGD	60:20:20	ALL-IDB1 + ALL-IDB2 + Barts	657	394	–	131	132
ViT1	Augmentation (8 scenarios) No k-fold cross-validation		60:20:20			394	3152	131	
ViT2	Augmentation (8 scenarios) K-fold cross-validation		Training-validation (80%) and testing (20%) K-fold splitting for training-validation (4 folds)			394*	3152*	131*	
ViT3	Augmentation (8 scenarios) K-fold cross-validation Gaussian pyramid (original images only)		Training-validation (80%) and testing (20%) K-fold splitting for training-validation (4 folds)			394*	3940*	131*	
ViT4	Augmentation (8 scenarios) K-fold cross-validation Gaussian pyramid (original and augmented images)		Training-validation (80%) and testing (20%) K-fold splitting for training-validation (4 folds)			394*	9456*	131*

**Table 4:** Configurations and dataset splits for external validation experiments. The experiments include ViT models with no augmentation, with augmentation, and with k-fold cross-validation (4 folds). Additionally, Gaussian pyramid transformations are applied in some cases. The datasets used include ALL-IDB1, ALL-IDB2, Barts, and combinations of them. The training, validation, and testing sizes are specified for each experiment. The asterisk (*) in the tables indicates that one fold in the 4-fold cross-validation resulted in different training and validation sizes compared to the other folds.

Label	Description	Optimization algorithm	Training/Validation split ratio	Training/Validation dataset	Total dataset Size	Training set size	Augmented training set size	Validation set size	Testing dataset	Testing set size
ViT0	No augmentation	Adam/SGD	80:20	ALL-IDB1	108	86	–	22	ALL-IDB2	260
									Barts	289
									Barts + ALL-IDB2	549
				ALL-IDB2	260	208	–	52	ALL-IDB1	108
									Barts	289
									Barts + ALL-IDB1	397
				Barts	289	231	–	58	ALL-IDB1	108
									ALL-IDB2	260
				ALL-IDB1 + ALL-IDB2	368	294	–	74	Barts	289
ViT1	Augmentation (8 scenarios)	Adam/SGD	80:20	ALL-IDB1	108	86	688	22	ALL-IDB2	260
									Barts	289
									Barts + ALL-IDB2	549
				ALL-IDB2	260	208	1664	52	ALL-IDB1	108
									Barts	289
									Barts + ALL-IDB1	397
				Barts	289	231	1848	58	ALL-IDB1	108
									ALL-IDB2	260
				ALL-IDB1 + ALL-IDB2	368	294	2352	74	Barts	289
ViT2	Augmentation (8 scenarios) K-fold cross-validation	Adam/SGD	K-fold splitting (4 folds)	ALL-IDB1	108	81	648	27	ALL-IDB2	260
									Barts	289
									Barts + ALL-IDB2	549
				ALL-IDB2	260	195	1560	65	ALL-IDB1	108
									Barts	289
									Barts + ALL-IDB1	397
				Barts	289	217*	1736*	72*	ALL-IDB1	108
									ALL-IDB2	260
				ALL-IDB1 + ALL-IDB2	368	276	2208	92	Barts	289
ViT3	Augmentation (8 scenarios) K-fold cross-validation Gaussian pyramid (original images only)	Adam/SGD	K-fold splitting (4 folds)	ALL-IDB1	108	81	810	27	ALL-IDB2	260
									Barts	289
									Barts + ALL-IDB2	549
				ALL-IDB2	260	195	1950	65	ALL-IDB1	108
									Barts	289
									Barts + ALL-IDB1	397
				Barts	289	217*	2170*	72*	ALL-IDB1	108
									ALL-IDB2	260
				ALL-IDB1 + ALL-IDB2	368	276	2760	92	Barts	289
ViT4	Augmentation (8 scenarios) K-fold cross-validation Gaussian pyramid (original and augmented images)	Adam/SGD	K-fold splitting (4 folds)	ALL-IDB1	108	81	1944	27	ALL-IDB2	260
									Barts	289
									Barts + ALL-IDB2	549
				ALL-IDB2	260	195	4680	65	ALL-IDB1	108
									Barts	289
									Barts + ALL-IDB1	397
				Barts	289	217*	5208*	72*	ALL-IDB1	108
									ALL-IDB2	260
				ALL-IDB1 + ALL-IDB2	368	276	6624	92	Barts	289

### 2.7 Performance evaluation

We evaluated the performance of the ViT models in the experiments described using various performance metrics. Training and validation losses were computed to monitor the models' performance during the training phase. Additional metrics like sensitivity, specificity, positive- and negative-likelihood ratios, positive-predictive value (PPV), and negative-predictive value (NPV) were also calculated. Overall model performance was further analyzed by calculating accuracy, miss rate, and precision. The area under the receiver operating characteristic curve (AUROC) was also computed to evaluate the model's ability in distinguishing between positive and negative cases and quantify its classification performance.

## 3 Results

The results highlight both the internal testing performance, focusing on the models' ability to optimize during training/validation, and the external testing, which tests their generalization to new datasets. Notable differences emerged between the models in terms of convergence speed and generalization capability, particularly across different datasets. The following sections present the results for internal and external validation.

### 3.1 Internal testing

**Fig. 2:**
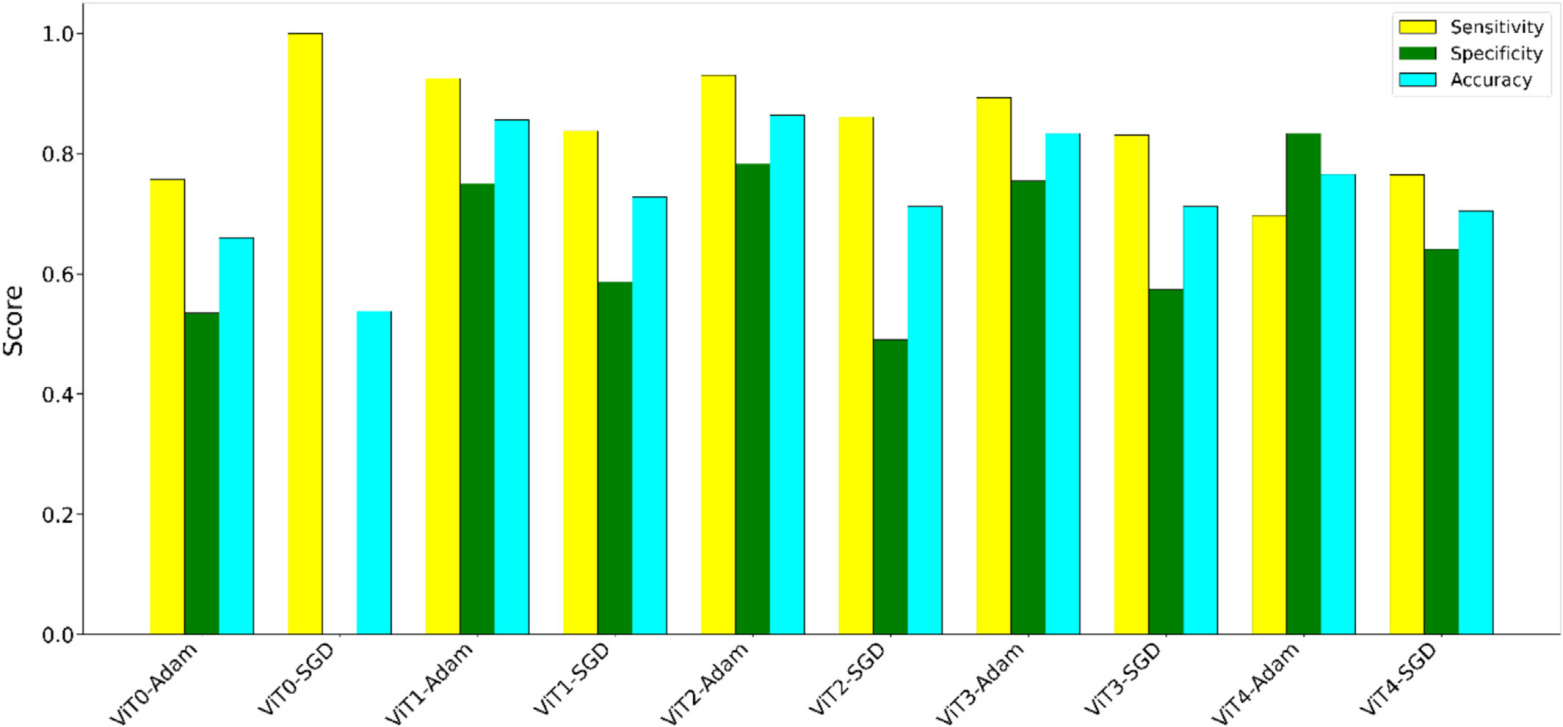
Performance comparison between various VIT models (ViT0–ViT4) optimized using either Adam or SGD algorithms. Models trained with Adam generally showed higher sensitivity (yellow), specificity (green), and accuracy (cyan) scores than models trained with SGD. The results highlight the differential impact of optimizers, data augmentation, and splitting on key performance metrics across model variations. (For interpretation of the references to color in this figure legend, the reader is referred to the web version of this article.)

Accuracy, specificity, and sensitivity across the ViT models trained with Adam and SGD optimizers are shown in Fig. 2. The models trained with Adam consistently outperformed those trained with SGD, showing higher accuracy, specificity, and sensitivity across most models, and highlighting Adam trained models' ability to correctly classify both positive (i.e., blasts are present) and negative cases. Models with data augmentation and advanced data splitting generally perform better than those without. The best-performing model, ViT2-Adam, achieved the highest accuracy of 0.86.

A detailed breakdown of the training and validation losses for all models (ViT0–ViT4) and optimization algorithms (Adam and SGD) is provided in Supplementary Table S1. The lowest validation loss of 0.437 was achieved with the ViT4-Adam model, which used data augmentation and k-fold cross-validation, whereas ViT0, which had no data augmentation and used an 80:20 split, performed the worst with the highest validation loss (0.675). This confirms that using a broader set of augmentations and cross-validation strategies improves model generalization. Models trained with the Adam optimizer consistently required fewer epochs to reach early stopping, indicating better convergence compared to those trained with SGD, which often reached the maximum epoch limit. Supplementary Fig. F1 shows the training and validation loss across epochs, highlighting the model (ViT4-Adam) with the best overall loss and its rapid convergence in contrast with the divergence between training and validation loss stopped after reaching the maximum number of epochs for both optimization algorithms for ViT4-SGD.

Adam's best performance is also confirmed in Supplementary Table S2, where it consistently outperforms SGD across all ViT models in terms of PPV and NPV.

**Fig. 3:**
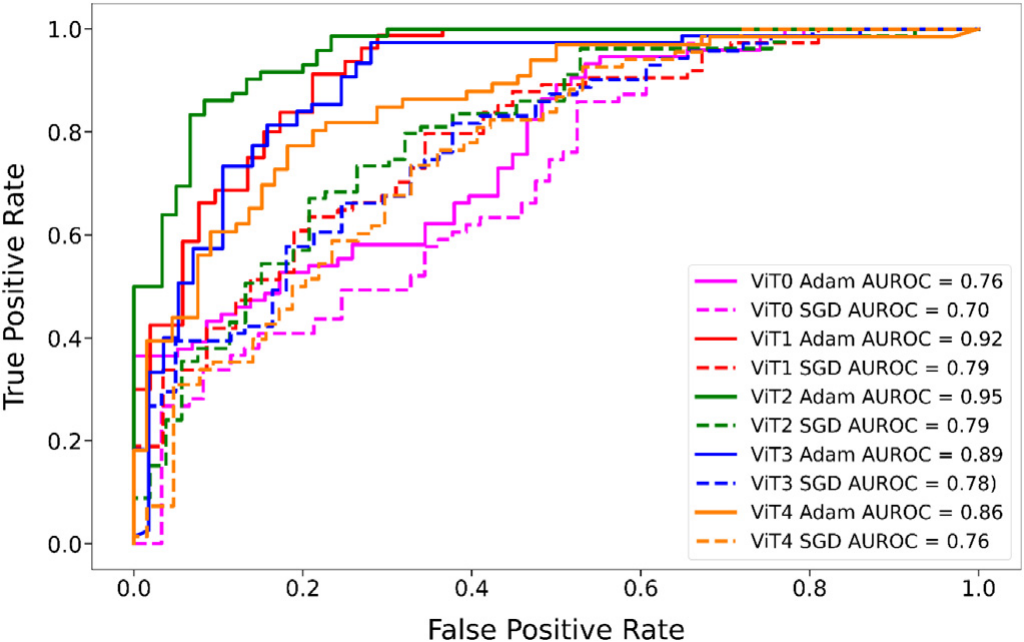
ROC curves comparing AUROC performance for ViT models across different configurations (ViT0–ViT4) and optimizers (Adam and SGD). Adam combined with data augmentation and other advanced techniques like K-fold cross-validation and Gaussian pyramids consistently achieves higher AUROC values.

**Fig. 4:**
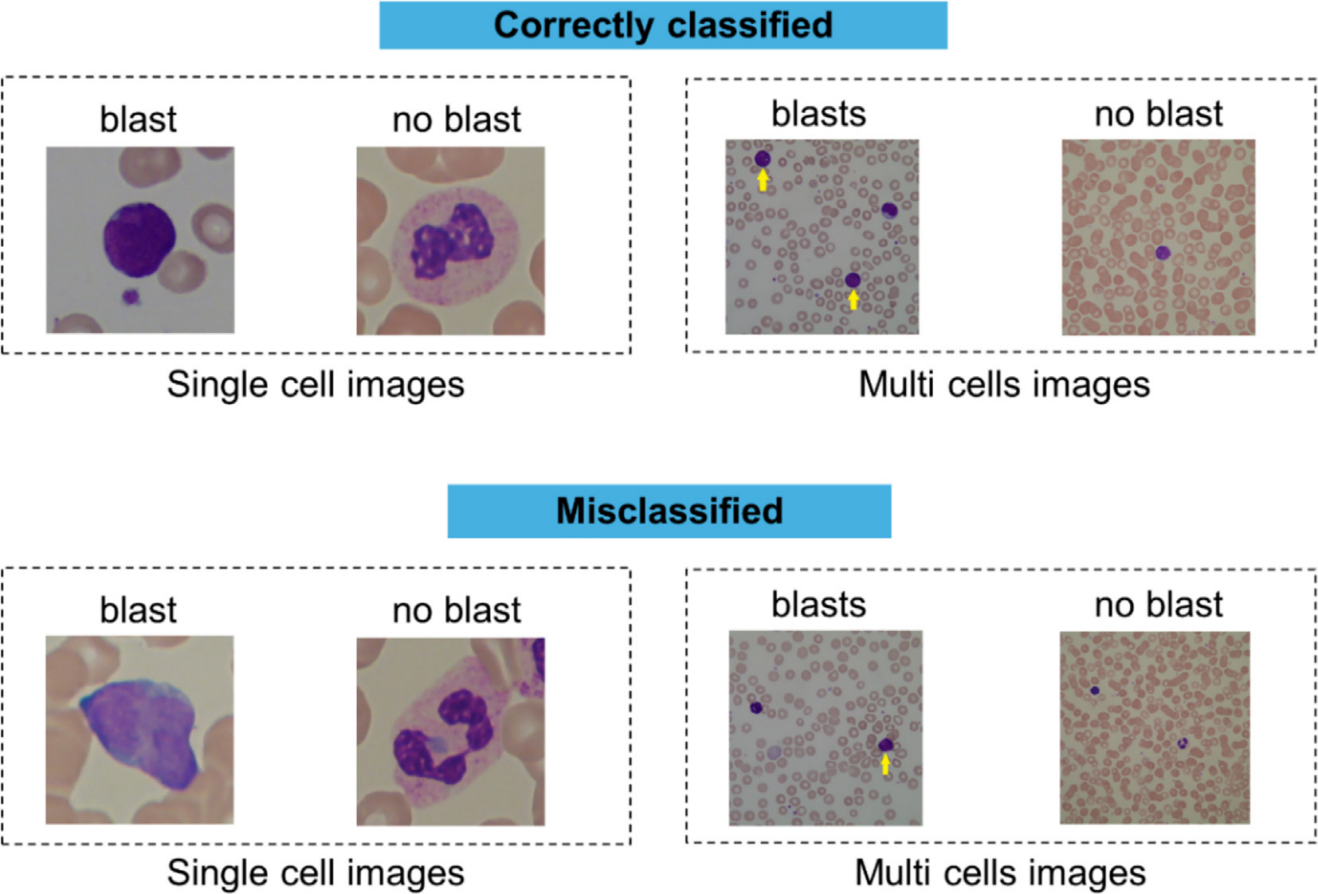
Examples of correctly classified and misclassified images by the best-performing model ViT2-Adam. Correctly classified images are showed at the top, whereas misclassified images are showed at the bottom. In the misclassified images, the model made incorrect predictions: images labeled as “blast” were classified as “no blast,” and images labeled as “no blast” were classified as “blast.” The yellow arrows indicate the blasts present in multicell images. (For interpretation of the references to color in this figure legend, the reader is referred to the web version of this article.)

Fig. 3 shows ROC curves on the test data along with the AUROC for each model, confirming that models optimized with Adam generally perform better than those with SGD across all ViT configurations. ViT2-Adam showed the highest AUROC (0.95), followed by ViT1-Adam (0.92) and ViT3-Adam (0.89). Examples of images that were correctly classified and misclassified by the best-performing model ViT2-Adam are shown in Fig. 4. Models trained with SGD resulted in worse performances, with ViT0-SGD showing the lowest AUROC (0.70) and poorest classification ability.

### 3.2 External testing

**Fig. 5:**
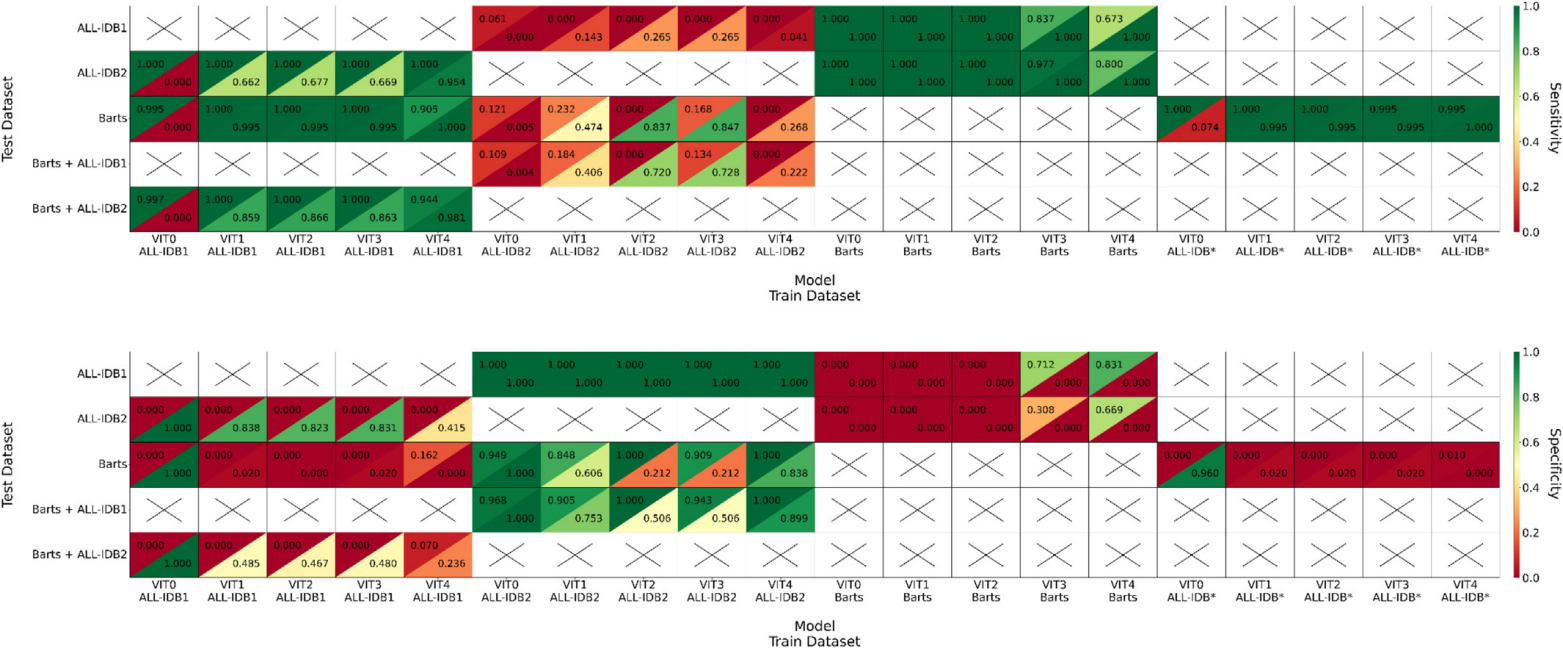
Sensitivity (top) and specificity (bottom) of various VIT models (ViT0–ViT4) trained/validated/tested using various datasets. The models are optimized using either Adam (upper triangle) or SGD (lower triangle). ALL-IDB* refers to the training/validation dataset including ALL-IDB1 and ALL-IDB2.

**Fig. 6:**
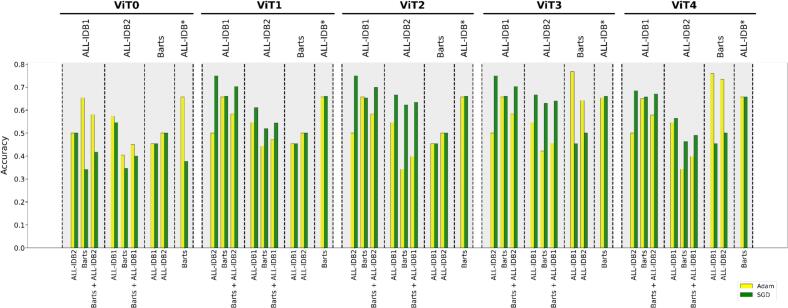
Accuracy values across different models (ViT0–ViT4) using Adam and SGD optimizers. The x-axis shows the training/validation datasets (top) and the corresponding testing datasets (bottom). ALL-IDB* refers to the training/validation dataset including ALL-IDB1 and ALL-IDB.

The performances in terms of specificity, sensitivity, and accuracy of the ViT models trained, validated, and tested on different datasets using Adam and SGD optimizers are shown in Fig. 5 and Fig. 6. Sensitivity performance across the various models and datasets varied significantly based on the use of augmentation, k-fold cross-validation, and dataset size. ViT models trained and validated on dataset such as ALL-IDB1, Barts, or a combination of ALL-IDB1 and ALL-IDB2 generally outperformed those trained and validated on ALL-IDB2 alone. For specificity, however, ViT models trained and validated on the database ALL-IDB2 showed superior performance compared to the other models.

Accuracy results further highlighted the impact of training data size and model complexity on the performance of the models. When training and validating on ALL-IDB1 and testing on ALL-IDB2, models using SGD with augmentation and k-fold cross-validation (ViT1–ViT4) showed higher accuracies (≥0.64) compared to models without these techniques, such as ViT0. When training on ALL-IDB2 and testing on Barts or ALL-IDB1 (as a standalone and combined), the performance of ViT2 and ViT3 models showed the best accuracy values (≥0.623). Models with Adam and Gaussian pyramid for both original and augmented images trained on Barts and tested on ALL-IDB1 or ALL-IDB2, outperformed the other model showing accuracies higher than 0.64. In particular, ViT3 and ViT4 model trained with Adam on Barts and tested on ALL-IDB1 showed the highest accuracies (≥0.759) overall, highlighting the benefit of dataset size in model generalization. Interestingly, the combination of multiple datasets for training (e.g., ALL-IDB1 + ALL-IDB2) generally led to similar accuracies for all ViT models with augmentation and k-fold cross-validation when tested on the Barts dataset.

The AUROC scores (Supplementary Fig. F2) show that the ViT0 models obtained the lowest performances across all dataset combinations, with sometimes drastic variation between the Adam and SGD optimizers. Similar performances are observed also with other models (ViT1–ViT4) with the only exception of models trained/validated with ALL-IDB1 dataset where the performance improved drastically. The best values of AUROC are obtained when training/validating the models with ALL-IDB2 dataset and testing them on ALL-IDB1 dataset or when training/validating them with Barts dataset and testing them on ALL-IDB1 or ALL-IDB2. For most models, it was also noted a general trend of Adam outperforming SGD when trained on the Barts dataset and SGD outperforming Adam when trained on ALL-IDB1.

A detailed breakdown of the training and validation losses, PPV, NPV, disease prevalence, and miss rate for all models (ViT0–ViT4) and optimization algorithms (Adam and SGD) is provided in Supplementary Tables S3 and S4. Training and validation loss over epochs, with examples of ViT models demonstrating the lowest overall loss, rapid convergence, reaching the maximum number of epochs during training, and divergence between training and validation loss, is shown in Supplementary Fig. F3.

## 4 Discussion

In this study, we systematically benchmarked ViT models for blast cell detection in digital blood films. Rather than proposing a novel architecture, our focus was on building and validating a practical and reproducible ViT-based pipeline, assessing how training strategies, data augmentation, and preprocessing impact performance across both public and clinical datasets. This benchmarking effort provides a pathway toward effective deployment of ViTs in real-world diagnostic settings. The main contributions and findings of this study can be summarized as follows: (a) a comprehensive benchmarking of ViT models with varying degrees of data augmentation, cross-validation, and Gaussian pyramid transformations; (b) extensive internal and external validation to evaluate generalizability using publicly available data repositories and a dataset from the Barts Health NHS Trust; (c) consistent superiority of the Adam optimizer over SGD in terms of convergence speed, accuracy, sensitivity, and specificity and (d) evidence that models incorporating data augmentation and Gaussian pyramid down sampling achieve improved generalization, particularly when combined with k-fold cross-validation.

### 4.1 Model performance, optimization, and preprocessing techniques

The results shown in this work highlight the need of preprocessing techniques before training the model as well as model validation techniques. As shown in many other works published in literature,^22^^,^^23^^,^^30^^,^^33^^,^^35^^,^^37^ data augmentation and cross-validation play an important role in improving the performance of the deep learning models. In our work, the model ViT0, the baseline ViT model with the standard train-test split or hold-out cross-validation and no data preprocessing techniques, consistently showed the lowest performance, with higher training and validation losses for all datasets. This proves that the model has very limited ability to generalize on unseen data when augmentation is not used. In contrast, ViT1–ViT4 models, where the size of the training set was increased applying different transformations, showed a lower validation loss and shorter training times. The best generalization ability, with consistently lower validation losses and higher AUROC values across internal and external testing datasets, was observed for ViT2 and ViT3 models, which used data augmentation, k-fold cross-validation, and Gaussian pyramid downsampling. Using k-fold cross-validation in models ViT2 and ViT3 improved stability and robustness, particularly for models trained on smaller datasets^27^ such as ALL-IDB1. Further improvements in performance were observed for the ViT3 model, where Gaussian pyramid transformations were included. However, this additional preprocessing technique resulted in longer training times, suggesting that the improvement in terms of performance is proportional to the computational cost.^14^^,^^32^ It is also interesting to note that the most complex model, ViT4, which uses data augmentation, k-fold cross-validation, and Gaussian pyramid transformations to both original and augmented images, did not perform better than the ViT3 model. This suggests that even if data augmentation might improve the generalization ability of the model, too many transformations may introduce noise rather than meaningful variability, especially when the dataset size is small. Notably, our best model (ViT2-Adam) achieved an accuracy of 86%, surpassing other models using the ALL-IDB dataset, such as the sine cosine algorithm based deep CNN model by Sneha and Alagu^28^ or the global–local attention Transformer model proposed by Chen et al.,^7^ which achieved an accuracy of 81% and 79.45%, respectively. Our best model showed better accuracies than the CNN, VGG16, or random forest based models in the work by Rezayi et al.^25^ that obtained accuracies of 82.1%, 84.62%, and 81.72%, respectively.

### 4.2 Impact of dataset size and composition

Our findings showed that the performance of the models was strongly influenced by the size and diversity of the dataset. In general, better performance was observed in ViT models trained and validated on multicell images most likely for the richer and diverse information they contain. For example, ViT models trained on Barts and the ALL-IDB1 datasets resulted in higher accuracies and AUROC scores compared to the ALL-IDB datasets. The model generalizability improved when a combined dataset was used (i.e., model trained with ALL-IDB1 + ALL-IDB2 and tested on the Barts dataset) further proving how training on diverse and heterogeneous datasets play a fundamental role for deep learning models.^3^^,^^31^

Models trained with the multicell images included in the ALL-IDB2 dataset resulted in a better specificity, but lower sensitivity compared to models trained on ALL-IDB1 or Barts datasets. This is probably because the ALL-IDB2 may contain less variability in the set of images containing no blasts. The direct consequence is that models that are more conservative in identifying blast cells might miss some cases where blasts are actually present. In contrast, the models trained on Barts or ALL-IDB1 dataset, which contains more diverse blood film images, achieved better sensitivity. This proves the importance of training on diverse clinical data to improve model generalization.^20^^,^^36^

### 4.3 Comparison of optimization algorithms

For all the experiments performed in this work, Adam consistently outperformed SGD in terms of accuracy, sensitivity, and specificity, especially with ViT models that integrated data augmentation and k-fold cross-validation. This can be explained by the fact that the Adam optimizer combines momentum and adaptive per-parameter learning rates to stabilize updates and accelerate convergence in high-dimensional parameter spaces. When Adam was used to train models on smaller datasets (e.g., ALL-IDB1), it tended to overfit more quickly because of the adaptive learning rate, which can amplify noise and lead to overfitting when the number of training samples is limited. On the contrary, SGD showed better stability in models trained on smaller and more homogeneous datasets due to its uniform update mechanism that yields to smoother convergence and better generalization in limited data settings.

Overall, the results from this study suggest that the choice of optimizer when training a model should depend on dataset characteristics such as size, variability, and noise level. Adam should be used for models trained on larger and more heterogeneous datasets, whereas when training using data that are small or homogeneous, SGD should be the preferred optimizer.

### 4.4 Limitations and future work

Although the results of this study are promising, several limitations should be noted. The datasets used, particularly ALL-IDB1 and ALL-IDB2, are relatively small, and while augmentation and cross-validation helped mitigate this issue, larger datasets are needed to further validate the model's robustness and generalization capabilities before it can be fully deployed for clinical use. To this aim, we are currently collecting a new, larger dataset from Barts Health NHS Trust to further validate the best model and then trial it in a clinical setting.

## 5 Conclusion

Our study investigated and proved the potential of ViT model for the automated detection of blast cells in blood films. We showed that data augmentation, k-fold cross-validation, and advanced image processing techniques such as Gaussian pyramids significantly improve the performances of the ViT models, especially for small training sets or heterogeneous and multicell image datasets. However, we also proved that these strategies do not always result in significantly better performances of the ViT models highlighting the importance of balancing model design, data splitting, optimization, and preprocessing techniques. We plan to conduct further research to validate the ViT models developed in this work, aiming to improve their computational efficiency, to generalize them across larger, more diverse populations, and ultimately to prepare them for real-world clinical use.

## Ethics approval and consent to participate

Ethical approval for this study was obtained from the Queen Mary University of London and Barts Health NHS Trust Joint Research Management Office (JRMO) reference number 156630.

## Funding sources

This research did not receive any specific grant from funding agencies in the public, commercial, or not-for-profit sectors.

## Declaration of competing interest

No conflicts of interest declared.

The authors declare that they have no known competing financial interests or personal relationships that could have appeared to influence the work reported in this paper.

## Data Availability

Relevant data supporting the key findings of this study are available within the article and its Supplementary Information files. All other datasets and source code for statistical analyses generated during this study are available from the corresponding authors upon reasonable request.

## Appendix A Supplementary data

Supplementary material Includes Table S1, Fig F1, Table S2, Fig F2, Table S3, Table S4, and Fig F3, providing additional information to support the results referenced in the main text. Supplementary material
