# Supplementary material for: BloodImage: Benchmarking vision transformers for blast detection in digital blood films using public and clinical datasets
Source: J Pathol Inform. 2025 Oct 31;19:100525. doi: 10.1016/j.jpi.2025.100525 (PMC12719678; doi:10.1016/j.jpi.2025.100525)

**BloodImage: Benchmarking Vision Transformers for Blast Detection in Digital Blood Films Using Public and Clinical Datasets**

Concetta Piazzese^1,2,3^, Sophie Williams^1,2,3^, Gregory Slabaugh^3^, Timothy Farren^4^, Tanya Freeman^5^, Laura Aiken^5^, Juswal Dadhra^4^, Stefan Browne^6^, Simon Deltadahl^7^, BloodCounts! Consortium, Suthesh Sivapalaratnam^2,3,5,8^

^1^ Barts Life Sciences, Barts Health NHS Trust, London, United Kingdom.

^2^ PHURI, Queen Mary University of London, London, United Kingdom.

^3^ DERI, Queen Mary University of London, London, United Kingdom.

^4^ NHS East and South East London (ESEL) Pathology Partnership, London, United Kingdom.

^5^ Clinical Haematology, Barts Health NHS Trust, London, United Kingdom.

^6^ Automation Consultants, Reading, United Kingdom.

^7^ Department of Applied Mathematics and Theoretical Physics, University of Cambridge, Cambridge, UK

^8^ Blizard, Queen Mary University of London, London, United Kingdom.

**Supplementary material**

**Supplementary material**

Table S1. **Overview of training and validation losses for all models (VIT0 to VIT4) and optimisation algorithms (Adam and SGD).**

| **Label** | **Optimisation algorithm** | **Fold** | **Traininig/Validation time (HH:MM:SS)** | **Training mean loss** | **Validation mean loss** | **Training loss (last epoch)** | **Training loss (best epoch)** | **Validation loss (last epoch)** | **Validation loss (best epoch)** | **Best epoch** | **Last epoch** | **Reason** |
| --- | --- | --- | --- | --- | --- | --- | --- | --- | --- | --- | --- | --- |
| ViT0 | Adam | - | 00:30:07 | 0.604 | 0.596 | 0.577 | 0.583 | 0.565 | 0.562 | 45 | 55 | Early stopping due to no improvement in validation loss at epoch 55 |
|  | SGD | - | 00:49:54 | 0.685 | 0.675 | 0.680 | 0.680 | 0.667 | 0.667 | 98 | 100 | Stop due to maximum number of epochs reached |
| ViT1 | Adam | - | 03:27:43 | 0.552 | 0.506 | 0.464 | 0.476 | 0.423 | 0.422 | 47 | 57 | Early stopping due to no improvement in validation loss at epoch 57 |
|  | SGD | - | 05:22:39 | 0.645 | 0.648 | 0.612 | 0.616 | 0.627 | 0.626 | 91 | 100 | Stop due to maximum number of epochs reached |
| ViT2 | Adam | 1 | 13:25:05 | 0.478 | 0.468 | 0.453 | 0.467 | 0.450 | 0.442 | 57 | 67 | Early stopping due to no improvement in validation loss at epoch 67 |
|  |  | 2 |  |  |  | 0.447 | 0.463 | 0.470 | 0.467 | 51 | 61 | Early stopping due to no improvement in validation loss at epoch 61 |
|  |  | 3 |  |  |  | 0.452 | 0.477 | 0.507 | 0.483 | 32 | 42 | Early stopping due to no improvement in validation loss at epoch 42 |
|  |  | 4 |  |  |  | 0.481 | 0.505 | 0.496 | 0.481 | 23 | 33 | Early stopping due to no improvement in validation loss at epoch 33 |
| ViT2 | SGD | 1 | 22:54:52 | 0.622 | 0.619 | 0.624 | 0.624 | 0.616 | 0.614 | 99 | 100 | Stop due to maximum number of epochs reached |
|  |  | 2 |  |  |  | 0.622 | 0.622 | 0.620 | 0.620 | 100 | 100 | Stop due to maximum number of epochs reached |
|  |  | 3 |  |  |  | 0.627 | 0.627 | 0.603 | 0.603 | 99 | 100 | Stop due to maximum number of epochs reached |
|  |  | 4 |  |  |  | 0.613 | 0.613 | 0.638 | 0.638 | 99 | 100 | Stop due to maximum number of epochs reached |
| ViT3 | Adam | 1 | 14:33:38 | 0.472 | 0.460 | 0.458 | 0.484 | 0.448 | 0.446 | 33 | 43 | Early stopping due to no improvement in validation loss at epoch 43 |
|  |  | 2 |  |  |  | 0.458 | 0.469 | 0.427 | 0.422 | 40 | 50 | Early stopping due to no improvement in validation loss at epoch 50 |
|  |  | 3 |  |  |  | 0.443 | 0.454 | 0.496 | 0.457 | 39 | 49 | Early stopping due to no improvement in validation loss at epoch 49 |
|  |  | 4 |  |  |  | 0.470 | 0.480 | 0.528 | 0.514 | 34 | 44 | Early stopping due to no improvement in validation loss at epoch 44 |
| ViT3 | SGD | 1 | 04:09:52 | 0.609 | 0.607 | 0.609 | 0.609 | 0.606 | 0.603 | 99 | 100 | Stop due to maximum number of epochs reached |
|  |  | 2 |  |  |  | 0.607 | 0.607 | 0.604 | 0.604 | 100 | 100 | Stop due to maximum number of epochs reached |
|  |  | 3 |  |  |  | 0.610 | 0.610 | 0.618 | 0.618 | 100 | 100 | Stop due to maximum number of epochs reached |
|  |  | 4 |  |  |  | 0.607 | 0.610 | 0.607 | 0.605 | 95 | 100 | Stop due to maximum number of epochs reached |
| ViT4 | Adam | 1 | 22:12:52 | 0.450 | 0.437 | 0.439 | 0.452 | 0.425 | 0.408 | 21 | 31 | Early stopping due to no improvement in validation loss at epoch 31 |
|  |  | 2 |  |  |  | 0.431 | 0.443 | 0.454 | 0.443 | 24 | 34 | Early stopping due to no improvement in validation loss at epoch 34 |
|  |  | 3 |  |  |  | 0.430 | 0.448 | 0.465 | 0.455 | 16 | 26 | Early stopping due to no improvement in validation loss at epoch 26 |
|  |  | 4 |  |  |  | 0.442 | 0.457 | 0.444 | 0.442 | 14 | 24 | Early stopping due to no improvement in validation loss at epoch 24 |
| ViT4 | SGD | 1 | 20:03:52 | 0.583 | 0.580 | 0.592 | 0.592 | 0.555 | 0.552 | 99 | 100 | Stop due to maximum number of epochs reached |
|  |  | 2 |  |  |  | 0.582 | 0.582 | 0.570 | 0.570 | 100 | 100 | Stop due to maximum number of epochs reached |
|  |  | 3 |  |  |  | 0.566 | 0.571 | 0.637 | 0.632 | 68 | 78 | Early stopping due to no improvement in validation loss at epoch 78 |
|  |  | 4 |  |  |  | 0.586 | 0.586 | 0.564 | 0.564 | 99 | 100 | Stop due to maximum number of epochs reached |


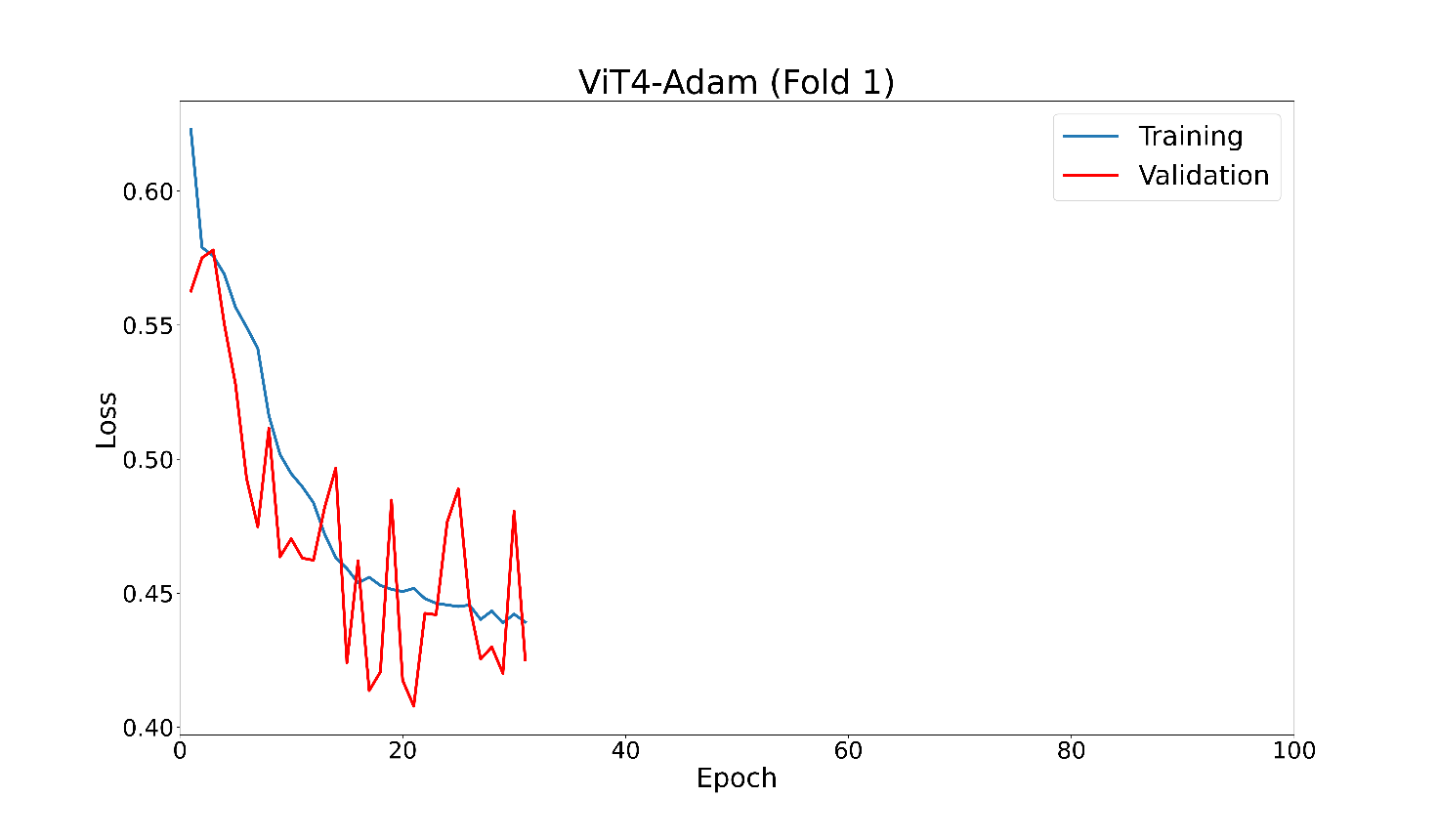


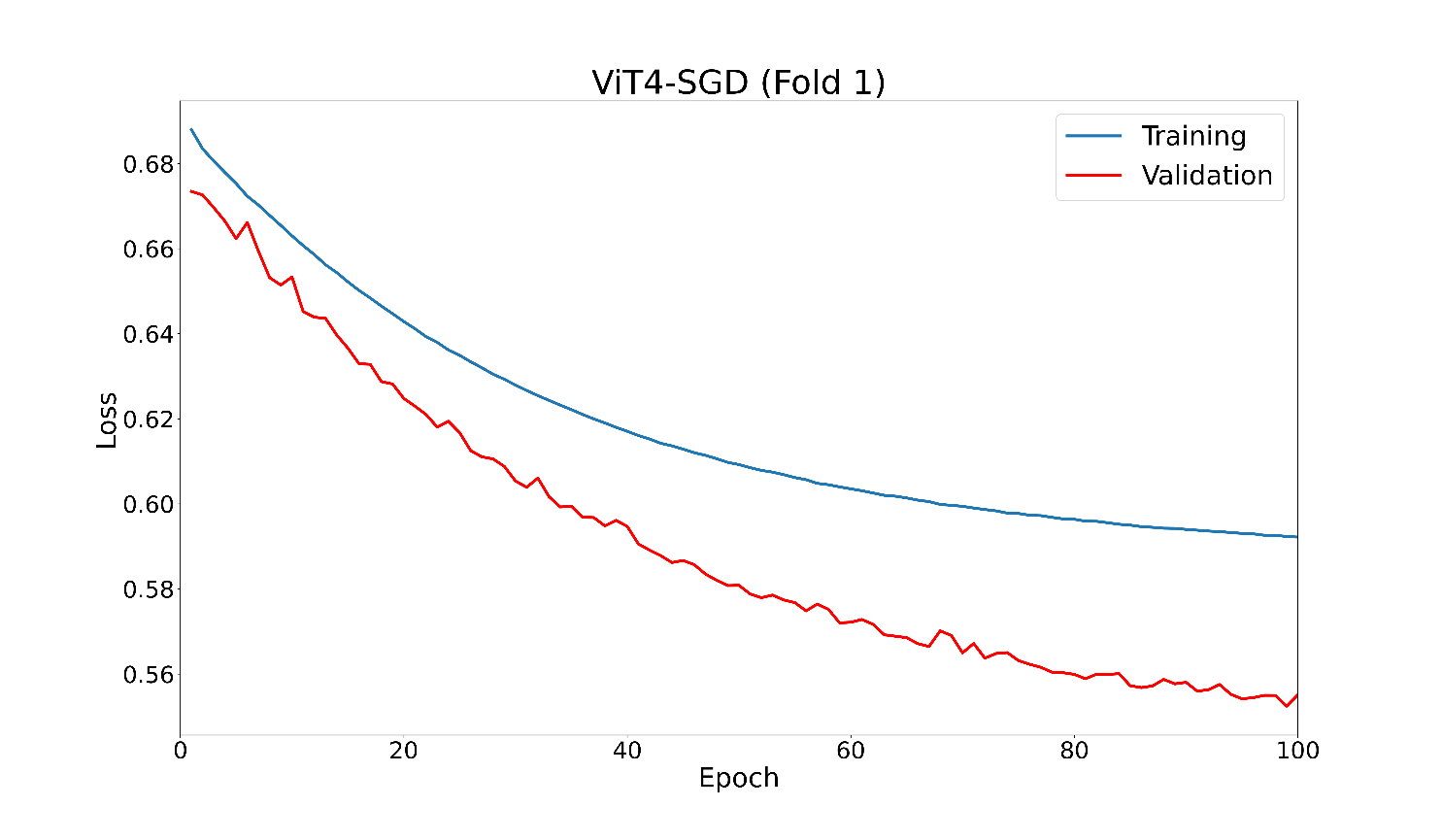


Figure F1. **Training and validation loss across epochs of two different models.** The rapid convergence of the VIT4-Adam model, with the lowest overall loss, is shown at the top of the figure, while the divergence observed in the VIT4-SGD model is shown at the bottom of the figure, with the model being stopped after reaching the maximum number of epochs

Table S2. **Performance metrics for ViT models during internal testing.** PPV, NPV, disease prevalence, and miss rate are shown for ViT models (ViT0 to ViT4) optimized using Adam and SGD algorithms. PPV, positive predictive value; NPV, negative predictive value.

| **Label** | **Optimization algorithm** | **PPV** | **NPV** | **Disease prevalence** | **Miss rate** |
| --- | --- | --- | --- | --- | --- |
| ViT0 | Adam | 0.675 | 0.633 | 0.561 | 0.243 |
|  | SGD | 0.538 | N/A | 0.538 | 0.000 |
| ViT1 | Adam | 0.851 | 0.867 | 0.606 | 0.075 |
|  | SGD | 0.721 | 0.739 | 0.561 | 0.162 |
| ViT2 | Adam | 0.838 | 0.904 | 0.545 | 0.069 |
|  | SGD | 0.716 | 0.703 | 0.598 | 0.139 |
| ViT3 | Adam | 0.827 | 0.843 | 0.568 | 0.107 |
|  | SGD | 0.694 | 0.745 | 0.538 | 0.169 |
| ViT4 | Adam | 0.807 | 0.733 | 0.500 | 0.303 |
|  | SGD | 0.693 | 0.719 | 0.515 | 0.235 |


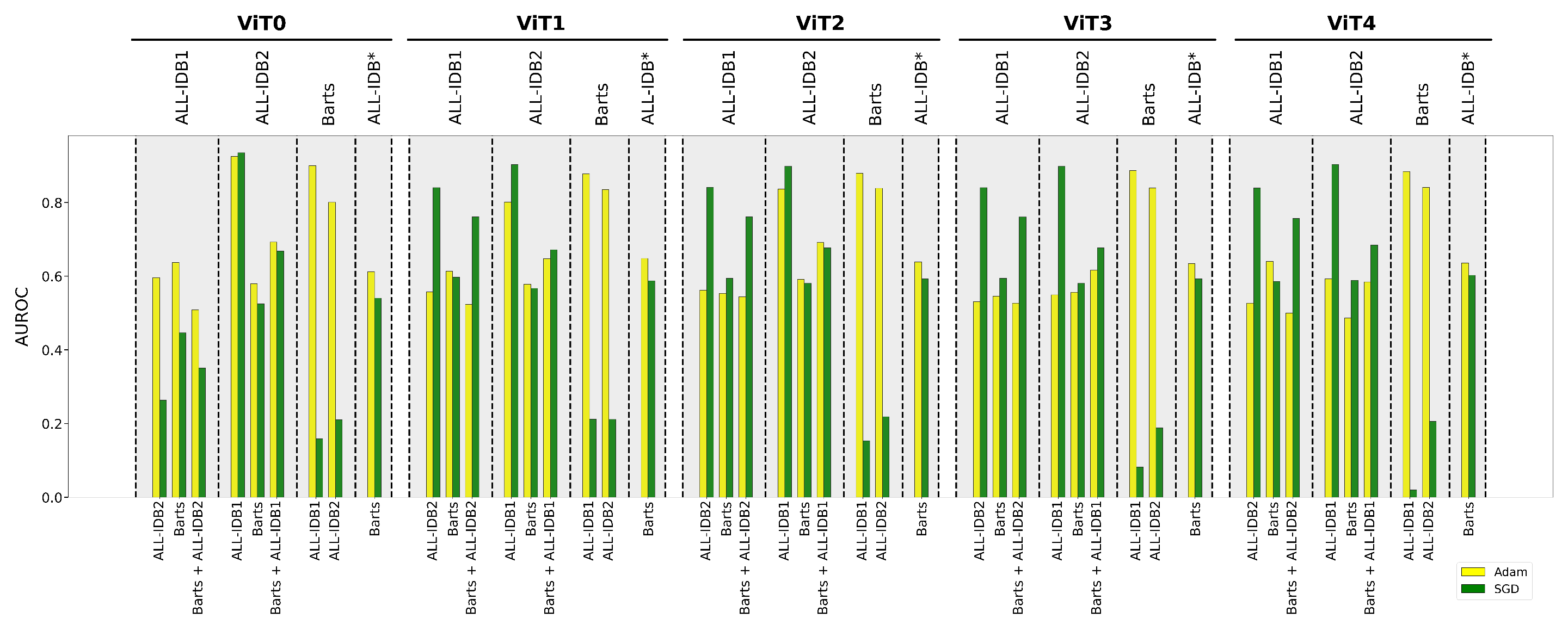


Figure F2. **AUROC values for various models (ViT0-ViT4) using the Adam and SGD optimisers. The** x-axis shows the training/validation datasets (top) alongside the corresponding testing datasets (bottom). ALL-IDB*" refers to the combined ALL-IDB1 and ALL-IDB2 datasets used for training and validation.

Table S3. **Summary of training and validation losses across all models (ViT0 to ViT4) and optimisation algorithms (Adam and SGD).**

| **Label** | **Traininig/Validation data** | **Optimisation algorithm** | **Fold** | **Traininig/Validation time (HH:MM:SS)** | **Training mean loss** | **Validation mean loss** | **Training loss (last epoch)** | **Training loss (best epoch)** | **Validation loss (last epoch)** | **Validation loss (best epoch)** | **Best epoch** | **Last epoch** | **Reason** |
| --- | --- | --- | --- | --- | --- | --- | --- | --- | --- | --- | --- | --- | --- |
| VIT0 | ALL-IDB1 | Adam | - | 00:11:42 | 0.471 | 0.465 | 0.314 | 0.314 | 0.314 | 0.314 | 100 | 100 | Stop due to maximum number of epochs reached |
|  |  | SGD | - | 00:01:09 | 0.693 | 0.706 | 0.692 | 0.694 | 0.707 | 0.705 | 1 | 11 | Early stopping due to no improvement in validation loss at epoch 11 |
|  | ALL-IDB2 | Adam | - | 00:07:11 | 0.597 | 0.606 | 0.539 | 0.568 | 0.572 | 0.563 | 16 | 26 | Early stopping due to no improvement in validation loss at epoch 26 |
|  |  | SGD | - | 00:24:46 | 0.689 | 0.695 | 0.681 | 0.681 | 0.687 | 0.687 | 100 | 100 | Stop due to maximum number of epochs reached |
|  | Barts | Adam | - | 00:07:58 | 0.648 | 0.666 | 0.651 | 0.646 | 0.670 | 0.655 | 16 | 26 | Early stopping due to no improvement in validation loss at epoch 26 |
|  |  | SGD | - | 00:10:37 | 0.647 | 0.647 | 0.643 | 0.643 | 0.644 | 0.644 | 27 | 37 | Early stopping due to no improvement in validation loss at epoch 37 |
|  | ALL-IDB1 + ALL-IDB2 | Adam | - | 00:38:43 | 0.540 | 0.524 | 0.495 | 0.495 | 0.419 | 0.419 | 100 | 100 | Stop due to maximum number of epochs reached |
|  |  | SGD | - | 00:34:52 | 0.685 | 0.688 | 0.673 | 0.673 | 0.677 | 0.676 | 99 | 100 | Stop due to maximum number of epochs reached |
| VIT1 | ALL-IDB1 | Adam | - | 00:30:47 | 0.398 | 0.398 | 0.313 | 0.314 | 0.343 | 0.314 | 28 | 38 | Early stopping due to no improvement in validation loss at epoch 38 |
|  |  | SGD | - | 01:13:10 | 0.666 | 0.665 | 0.642 | 0.642 | 0.642 | 0.642 | 100 | 100 | Stop due to maximum number of epochs reached |
|  | ALL-IDB2 | Adam | - | 01:32:18 | 0.423 | 0.440 | 0.342 | 0.342 | 0.339 | 0.320 | 37 | 47 | Early stopping due to no improvement in validation loss at epoch 47 |
|  |  | SGD | - | 02:52:42 | 0.655 | 0.630 | 0.626 | 0.626 | 0.582 | 0.582 | 100 | 100 | Stop due to maximum number of epochs reached |
|  | Barts | Adam | - | 00:29:10 | 0.653 | 0.628 | 0.649 | 0.658 | 0.622 | 0.620 | 3 | 13 | Early stopping due to no improvement in validation loss at epoch 13 |
|  |  | SGD | - | 00:51:47 | 0.627 | 0.716 | 0.627 | 0.627 | 0.714 | 0.713 | 16 | 26 | Early stopping due to no improvement in validation loss at epoch 26 |
|  | ALL-IDB1 + ALL-IDB2 | Adam | - | 01:47:06 | 0.439 | 0.430 | 0.343 | 0.358 | 0.355 | 0.343 | 28 | 38 | Early stopping due to no improvement in validation loss at epoch 38 |
|  |  | SGD | - | 04:05:30 | 0.643 | 0.625 | 0.604 | 0.604 | 0.573 | 0.572 | 99 | 100 | Stop due to maximum number of epochs reached |
| VIT2 | ALL-IDB1 | Adam | 1 | 02:24:16 | 0.316 | 0.315 | 0.313 | 0.313 | 0.313 | 0.313 | 50 | 60 | Early stopping due to no improvement in validation loss at epoch 60 |
|  |  |  | 2 |  |  |  | 0.313 | 0.314 | 0.330 | 0.319 | 25 | 35 | Early stopping due to no improvement in validation loss at epoch 35 |
|  |  |  | 3 |  |  |  | 0.316 | 0.315 | 0.330 | 0.313 | 43 | 53 | Early stopping due to no improvement in validation loss at epoch 53 |
|  |  |  | 4 |  |  |  | 0.313 | 0.324 | 0.313 | 0.313 | 25 | 35 | Early stopping due to no improvement in validation loss at epoch 35 |
|  |  | SGD | 1 | 03:44:11 | 0.656 | 0.659 | 0.640 | 0.641 | 0.709 | 0.705 | 98 | 100 | Stop due to maximum number of epochs reached |
|  |  |  | 2 |  |  |  | 0.638 | 0.638 | 0.623 | 0.623 | 100 | 100 | Stop due to maximum number of epochs reached |
|  |  |  | 3 |  |  |  | 0.679 | 0.697 | 0.671 | 0.665 | 4 | 14 | Early stopping due to no improvement in validation loss at epoch 14 |
|  |  |  | 4 |  |  |  | 0.647 | 0.648 | 0.646 | 0.645 | 96 | 100 | Stop due to maximum number of epochs reached |
|  | ALL-IDB2 | Adam | 1 | 03:01:38 | 0.512 | 0.500 | 0.541 | 0.548 | 0.540 | 0.528 | 9 | 19 | Early stopping due to no improvement in validation loss at epoch 19 |
|  |  |  | 2 |  |  |  | 0.515 | 0.604 | 0.636 | 0.580 | 2 | 12 | Early stopping due to no improvement in validation loss at epoch 12 |
|  |  |  | 3 |  |  |  | 0.340 | 0.339 | 0.346 | 0.342 | 42 | 52 | Early stopping due to no improvement in validation loss at epoch 52 |
|  |  |  | 4 |  |  |  | 0.543 | 0.558 | 0.551 | 0.550 | 4 | 14 | Early stopping due to no improvement in validation loss at epoch 14 |
|  |  | SGD | 1 | 11:18:59 | 0.613 | 0.611 | 0.622 | 0.622 | 0.605 | 0.604 | 99 | 100 | Stop due to maximum number of epochs reached |
|  |  |  | 2 |  |  |  | 0.605 | 0.605 | 0.627 | 0.625 | 99 | 100 | Stop due to maximum number of epochs reached |
|  |  |  | 3 |  |  |  | 0.599 | 0.599 | 0.612 | 0.611 | 99 | 100 | Stop due to maximum number of epochs reached |
|  |  |  | 4 |  |  |  | 0.624 | 0.624 | 0.603 | 0.603 | 100 | 100 | Stop due to maximum number of epochs reached |
|  | Barts | Adam | 1 | 04:17:25 | 0.641 | 0.650 | 0.609 | 0.609 | 0.789 | 0.780 | 1 | 11 | Early stopping due to no improvement in validation loss at epoch 11 |
|  |  |  | 2 |  |  |  | 0.634 | 0.639 | 0.652 | 0.625 | 51 | 61 | Early stopping due to no improvement in validation loss at epoch 61 |
|  |  |  | 3 |  |  |  | 0.658 | 0.657 | 0.632 | 0.590 | 13 | 23 | Early stopping due to no improvement in validation loss at epoch 23 |
|  |  |  | 4 |  |  |  | 0.656 | 0.656 | 0.604 | 0.603 | 15 | 25 | Early stopping due to no improvement in validation loss at epoch 25 |
|  |  | SGD | 1 | 01:50:19 | 0.653 | 0.644 | 0.655 | 0.655 | 0.611 | 0.611 | 9 | 19 | Early stopping due to no improvement in validation loss at epoch 19 |
|  |  |  | 2 |  |  |  | 0.657 | 0.657 | 0.605 | 0.603 | 3 | 13 | Early stopping due to no improvement in validation loss at epoch 13 |
|  |  |  | 3 |  |  |  | 0.645 | 0.645 | 0.638 | 0.638 | 4 | 14 | Early stopping due to no improvement in validation loss at epoch 14 |
|  |  |  | 4 |  |  |  | 0.611 | 0.656 | 0.761 | 0.724 | 1 | 11 | Early stopping due to no improvement in validation loss at epoch 11 |
|  | ALL-IDB1 + ALL-IDB2 | Adam | 1 | 09:08:56 | 0.371 | 0.361 | 0.382 | 0.384 | 0.392 | 0.355 | 38 | 48 | Early stopping due to no improvement in validation loss at epoch 48 |
|  |  |  | 2 |  |  |  | 0.381 | 0.384 | 0.390 | 0.370 | 33 | 43 | Early stopping due to no improvement in validation loss at epoch 43 |
|  |  |  | 3 |  |  |  | 0.362 | 0.342 | 0.565 | 0.337 | 41 | 51 | Early stopping due to no improvement in validation loss at epoch 51 |
|  |  |  | 4 |  |  |  | 0.361 | 0.375 | 0.391 | 0.382 | 59 | 69 | Early stopping due to no improvement in validation loss at epoch 69 |
|  |  | SGD | 1 | 15:50:41 | 0.596 | 0.591 | 0.587 | 0.587 | 0.603 | 0.603 | 100 | 100 | Stop due to maximum number of epochs reached |
|  |  |  | 2 |  |  |  | 0.596 | 0.597 | 0.602 | 0.598 | 99 | 100 | Stop due to maximum number of epochs reached |
|  |  |  | 3 |  |  |  | 0.598 | 0.600 | 0.576 | 0.576 | 97 | 100 | Stop due to maximum number of epochs reached |
|  |  |  | 4 |  |  |  | 0.599 | 0.600 | 0.587 | 0.587 | 99 | 100 | Stop due to maximum number of epochs reached |
| VIT3 | ALL-IDB1 | Adam | 1 | 03:32:02 | 0.321 | 0.313 | 0.313 | 0.313 | 0.313 | 0.313 | 50 | 60 | Early stopping due to no improvement in validation loss at epoch 60 |
|  |  |  | 2 |  |  |  | 0.313 | 0.341 | 0.325 | 0.313 | 29 | 39 | Early stopping due to no improvement in validation loss at epoch 39 |
|  |  |  | 3 |  |  |  | 0.313 | 0.316 | 0.314 | 0.313 | 44 | 54 | Early stopping due to no improvement in validation loss at epoch 54 |
|  |  |  | 4 |  |  |  | 0.315 | 0.315 | 0.313 | 0.313 | 48 | 58 | Early stopping due to no improvement in validation loss at epoch 58 |
|  |  | SGD | 1 | 04:39:03 | 0.654 | 0.643 | 0.689 | 0.714 | 0.682 | 0.678 | 1 | 11 | Early stopping due to no improvement in validation loss at epoch 11 |
|  |  |  | 2 |  |  |  | 0.632 | 0.632 | 0.628 | 0.628 | 100 | 100 | Stop due to maximum number of epochs reached |
|  |  |  | 3 |  |  |  | 0.631 | 0.631 | 0.645 | 0.645 | 100 | 100 | Stop due to maximum number of epochs reached |
|  |  |  | 4 |  |  |  | 0.641 | 0.641 | 0.620 | 0.620 | 100 | 100 | Stop due to maximum number of epochs reached |
|  | ALL-IDB2 | Adam | 1 | 09:34:18 | 0.343 | 0.333 | 0.355 | 0.345 | 0.329 | 0.329 | 54 | 64 | Early stopping due to no improvement in validation loss at epoch 64 |
|  |  |  | 2 |  |  |  | 0.332 | 0.340 | 0.360 | 0.359 | 46 | 56 | Early stopping due to no improvement in validation loss at epoch 56 |
|  |  |  | 3 |  |  |  | 0.335 | 0.359 | 0.314 | 0.313 | 47 | 57 | Early stopping due to no improvement in validation loss at epoch 57 |
|  |  |  | 4 |  |  |  | 0.324 | 0.326 | 0.333 | 0.330 | 54 | 64 | Early stopping due to no improvement in validation loss at epoch 64 |
|  |  | SGD | 1 | 14:05:42 | 0.599 | 0.597 | 0.600 | 0.600 | 0.600 | 0.600 | 100 | 100 | Stop due to maximum number of epochs reached |
|  |  |  | 2 |  |  |  | 0.592 | 0.592 | 0.610 | 0.610 | 100 | 100 | Stop due to maximum number of epochs reached |
|  |  |  | 3 |  |  |  | 0.590 | 0.590 | 0.593 | 0.593 | 100 | 100 | Stop due to maximum number of epochs reached |
|  |  |  | 4 |  |  |  | 0.613 | 0.613 | 0.584 | 0.584 | 100 | 100 | Stop due to maximum number of epochs reached |
|  | Barts | Adam | 1 | 14:34:58 | 0.617 | 0.637 | 0.635 | 0.637 | 0.667 | 0.658 | 20 | 30 | Early stopping due to no improvement in validation loss at epoch 30 |
|  |  |  | 2 |  |  |  | 0.611 | 0.612 | 0.631 | 0.631 | 99 | 100 | Stop due to maximum number of epochs reached |
|  |  |  | 3 |  |  |  | 0.612 | 0.612 | 0.629 | 0.629 | 100 | 100 | Stop due to maximum number of epochs reached |
|  |  |  | 4 |  |  |  | 0.607 | 0.608 | 0.631 | 0.630 | 99 | 100 | Stop due to maximum number of epochs reached |
|  |  | SGD | 1 | 02:07:31 | 0.657 | 0.648 | 0.627 | 0.640 | 0.697 | 0.686 | 1 | 11 | Early stopping due to no improvement in validation loss at epoch 11 |
|  |  |  | 2 |  |  |  | 0.661 | 0.661 | 0.599 | 0.593 | 6 | 16 | Early stopping due to no improvement in validation loss at epoch 16 |
|  |  |  | 3 |  |  |  | 0.615 | 0.665 | 0.746 | 0.719 | 2 | 12 | Early stopping due to no improvement in validation loss at epoch 12 |
|  |  |  | 4 |  |  |  | 0.662 | 0.662 | 0.595 | 0.593 | 4 | 14 | Early stopping due to no improvement in validation loss at epoch 14 |
|  | ALL-IDB1 + ALL-IDB2 | Adam | 1 | 11:40:45 | 0.336 | 0.327 | 0.327 | 0.333 | 0.337 | 0.318 | 38 | 48 | Early stopping due to no improvement in validation loss at epoch 48 |
|  |  |  | 2 |  |  |  | 0.327 | 0.337 | 0.338 | 0.319 | 47 | 57 | Early stopping due to no improvement in validation loss at epoch 57 |
|  |  |  | 3 |  |  |  | 0.335 | 0.336 | 0.354 | 0.330 | 41 | 51 | Early stopping due to no improvement in validation loss at epoch 51 |
|  |  |  | 4 |  |  |  | 0.322 | 0.336 | 0.364 | 0.343 | 39 | 49 | Early stopping due to no improvement in validation loss at epoch 49 |
|  |  | SGD | 1 | 19:50:40 | 0.582 | 0.578 | 0.590 | 0.590 | 0.561 | 0.561 | 100 | 100 | Stop due to maximum number of epochs reached |
|  |  |  | 2 |  |  |  | 0.575 | 0.576 | 0.601 | 0.599 | 98 | 100 | Stop due to maximum number of epochs reached |
|  |  |  | 3 |  |  |  | 0.578 | 0.578 | 0.584 | 0.584 | 100 | 100 | Stop due to maximum number of epochs reached |
|  |  |  | 4 |  |  |  | 0.586 | 0.586 | 0.568 | 0.568 | 100 | 100 | Stop due to maximum number of epochs reached |
| VIT4 | ALL-IDB1 | Adam | 1 | 03:51:06 | 0.321 | 0.322 | 0.313 | 0.328 | 0.325 | 0.317 | 6 | 16 | Early stopping due to no improvement in validation loss at epoch 16 |
|  |  |  | 2 |  |  |  | 0.316 | 0.324 | 0.387 | 0.345 | 8 | 18 | Early stopping due to no improvement in validation loss at epoch 18 |
|  |  |  | 3 |  |  |  | 0.313 | 0.313 | 0.313 | 0.313 | 29 | 39 | Early stopping due to no improvement in validation loss at epoch 39 |
|  |  |  | 4 |  |  |  | 0.319 | 0.318 | 0.340 | 0.313 | 15 | 25 | Early stopping due to no improvement in validation loss at epoch 25 |
|  |  | SGD | 1 | 13:55:14 | 0.568 | 0.567 | 0.578 | 0.578 | 0.575 | 0.575 | 100 | 100 | Stop due to maximum number of epochs reached |
|  |  |  | 2 |  |  |  | 0.543 | 0.543 | 0.577 | 0.577 | 100 | 100 | Stop due to maximum number of epochs reached |
|  |  |  | 3 |  |  |  | 0.576 | 0.577 | 0.548 | 0.545 | 99 | 100 | Stop due to maximum number of epochs reached |
|  |  |  | 4 |  |  |  | 0.575 | 0.575 | 0.569 | 0.569 | 100 | 100 | Stop due to maximum number of epochs reached |
|  | ALL-IDB2 | Adam | 1 | 13:31:26 | 0.343 | 0.331 | 0.331 | 0.350 | 0.328 | 0.313 | 23 | 33 | Early stopping due to no improvement in validation loss at epoch 33 |
|  |  |  | 2 |  |  |  | 0.337 | 0.341 | 0.360 | 0.332 | 36 | 46 | Early stopping due to no improvement in validation loss at epoch 46 |
|  |  |  | 3 |  |  |  | 0.338 | 0.354 | 0.374 | 0.332 | 16 | 26 | Early stopping due to no improvement in validation loss at epoch 26 |
|  |  |  | 4 |  |  |  | 0.320 | 0.329 | 0.358 | 0.346 | 25 | 35 | Early stopping due to no improvement in validation loss at epoch 35 |
|  |  | SGD | 1 | 33:04:31 | 0.556 | 0.555 | 0.578 | 0.578 | 0.519 | 0.519 | 100 | 100 | Stop due to maximum number of epochs reached |
|  |  |  | 2 |  |  |  | 0.548 | 0.548 | 0.570 | 0.570 | 100 | 100 | Stop due to maximum number of epochs reached |
|  |  |  | 3 |  |  |  | 0.539 | 0.539 | 0.587 | 0.587 | 100 | 100 | Stop due to maximum number of epochs reached |
|  |  |  | 4 |  |  |  | 0.559 | 0.560 | 0.546 | 0.543 | 98 | 100 | Stop due to maximum number of epochs reached |
|  | Barts | Adam | 1 | 12:57:02 | 0.637 | 0.633 | 0.596 | 0.614 | 0.625 | 0.616 | 55 | 65 | Early stopping due to no improvement in validation loss at epoch 65 |
|  |  |  | 2 |  |  |  | 0.636 | 0.640 | 0.664 | 0.652 | 11 | 21 | Early stopping due to no improvement in validation loss at epoch 21 |
|  |  |  | 3 |  |  |  | 0.654 | 0.665 | 0.659 | 0.591 | 1 | 11 | Early stopping due to no improvement in validation loss at epoch 11 |
|  |  |  | 4 |  |  |  | 0.625 | 0.629 | 0.698 | 0.672 | 18 | 28 | Early stopping due to no improvement in validation loss at epoch 28 |
|  |  | SGD | 1 | 05:06:12 | 0.672 | 0.644 | 0.640 | 0.644 | 0.652 | 0.652 | 1 | 11 | Early stopping due to no improvement in validation loss at epoch 11 |
|  |  |  | 2 |  |  |  | 0.656 | 0.656 | 0.612 | 0.604 | 12 | 22 | Early stopping due to no improvement in validation loss at epoch 22 |
|  |  |  | 3 |  |  |  | 0.640 | 0.740 | 0.656 | 0.655 | 1 | 11 | Early stopping due to no improvement in validation loss at epoch 11 |
|  |  |  | 4 |  |  |  | 0.635 | 0.647 | 0.666 | 0.663 | 1 | 11 | Early stopping due to no improvement in validation loss at epoch 11 |
|  | ALL-IDB1 + ALL-IDB2 | Adam | 1 | 21:11:25 | 0.326 | 0.330 | 0.320 | 0.321 | 0.367 | 0.334 | 31 | 41 | Early stopping due to no improvement in validation loss at epoch 41 |
|  |  |  | 2 |  |  |  | 0.319 | 0.326 | 0.330 | 0.324 | 32 | 42 | Early stopping due to no improvement in validation loss at epoch 42 |
|  |  |  | 3 |  |  |  | 0.330 | 0.334 | 0.347 | 0.339 | 25 | 35 | Early stopping due to no improvement in validation loss at epoch 35 |
|  |  |  | 4 |  |  |  | 0.323 | 0.321 | 0.325 | 0.322 | 29 | 39 | Early stopping due to no improvement in validation loss at epoch 39 |
|  |  | SGD | 1 | 46:35:35 | 0.545 | 0.542 | 0.534 | 0.537 | 0.562 | 0.555 | 94 | 100 | Stop due to maximum number of epochs reached |
|  |  |  | 2 |  |  |  | 0.547 | 0.547 | 0.536 | 0.536 | 100 | 100 | Stop due to maximum number of epochs reached |
|  |  |  | 3 |  |  |  | 0.562 | 0.562 | 0.511 | 0.511 | 100 | 100 | Stop due to maximum number of epochs reached |
|  |  |  | 4 |  |  |  | 0.532 | 0.533 | 0.568 | 0.567 | 99 | 100 | Stop due to maximum number of epochs reached |

Table S4. **Performance metrics, including PPV, NPV, disease prevalence, and miss rate, for ViT models (ViT0 to ViT4) optimised with Adam and SGD algorithms and tested externally.**

| **Label** | **Optimisation algorithm** | **Traininig/Validation data** | **Test data** | **Testing data size** | **PPV** | **NPV** | **Disease prevalence** | **Miss rate** |
| --- | --- | --- | --- | --- | --- | --- | --- | --- |
| VIT0 | Adam | ALL-IDB1 | ALL-IDB2 | 260 | 0.5 | N/A | 0.5 | 0 |
|  |  |  | Barts | 289 | 0.656 | 0 | 0.657 | 0.005 |
|  |  |  | Barts + ALL-IDB2 | 549 | 0.582 | 0 | 0.583 | 0.003 |
|  | SGD |  | ALL-IDB2 | 260 | N/A | 0.5 | 0.5 | 1 |
|  |  |  | Barts | 289 | N/A | 0.343 | 0.657 | 1 |
|  |  |  | Barts + ALL-IDB2 | 549 | N/A | 0.417 | 0.583 | 1 |
|  | Adam | ALL-IDB2 | ALL-IDB1 | 108 | 1 | 0.562 | 0.454 | 0.939 |
|  |  |  | Barts | 289 | 0.821 | 0.36 | 0.657 | 0.879 |
|  |  |  | Barts + ALL-IDB1 | 397 | 0.839 | 0.418 | 0.602 | 0.891 |
|  | SGD |  | ALL-IDB1 | 108 | N/A | 0.546 | 0.454 | 1 |
|  |  |  | Barts | 289 | 1 | 0.344 | 0.657 | 0.995 |
|  |  |  | Barts + ALL-IDB1 | 397 | 1 | 0.399 | 0.602 | 0.996 |
|  | Adam | Barts | ALL-IDB1 | 108 | 0.454 | N/A | 0.454 | 0 |
|  |  |  | ALL-IDB2 | 260 | 0.5 | N/A | 0.5 | 0 |
|  | SGD |  | ALL-IDB1 | 108 | 0.454 | N/A | 0.454 | 0 |
|  |  |  | ALL-IDB2 | 260 | 0.5 | N/A | 0.5 | 0 |
|  | Adam | ALL-IDB1 + ALL-IDB2 | Barts | 289 | 0.657 | N/A | 0.657 | 0 |
|  | SGD | ALL-IDB1 + ALL-IDB2 | Barts | 289 | 0.778 | 0.351 | 0.657 | 0.926 |
| VIT1 | Adam | ALL-IDB1 | ALL-IDB2 | 260 | 0.5 | N/A | 0.5 | 0 |
|  |  |  | Barts | 289 | 0.657 | N/A | 0.657 | 0 |
|  |  |  | Barts + ALL-IDB2 | 549 | 0.583 | N/A | 0.583 | 0 |
|  | SGD |  | ALL-IDB2 | 260 | 0.804 | 0.712 | 0.5 | 0.338 |
|  |  |  | Barts | 289 | 0.661 | 0.667 | 0.657 | 0.005 |
|  |  |  | Barts + ALL-IDB2 | 549 | 0.7 | 0.712 | 0.583 | 0.141 |
|  | Adam | ALL-IDB2 | ALL-IDB1 | 108 | N/A | 0.546 | 0.454 | 1 |
|  |  |  | Barts | 289 | 0.746 | 0.365 | 0.657 | 0.768 |
|  |  |  | Barts + ALL-IDB1 | 397 | 0.746 | 0.423 | 0.602 | 0.816 |
|  | SGD |  | ALL-IDB1 | 108 | 1 | 0.584 | 0.454 | 0.857 |
|  |  |  | Barts | 289 | 0.698 | 0.375 | 0.657 | 0.526 |
|  |  |  | Barts + ALL-IDB1 | 397 | 0.713 | 0.456 | 0.602 | 0.594 |
|  | Adam | Barts | ALL-IDB1 | 108 | 0.454 | N/A | 0.454 | 0 |
|  |  |  | ALL-IDB2 | 260 | 0.5 | N/A | 0.5 | 0 |
|  | SGD |  | ALL-IDB1 | 108 | 0.454 | N/A | 0.454 | 0 |
|  |  |  | ALL-IDB2 | 260 | 0.5 | N/A | 0.5 | 0 |
|  | Adam | ALL-IDB1 + ALL-IDB2 | Barts | 289 | 0.657 | N/A | 0.657 | 0 |
|  | SGD | ALL-IDB1 + ALL-IDB2 | Barts | 289 | 0.661 | 0.667 | 0.657 | 0.005 |
| VIT2 | Adam | ALL-IDB1 | ALL-IDB2 | 260 | 0.5 | N/A | 0.5 | 0 |
|  |  |  | Barts | 289 | 0.657 | N/A | 0.657 | 0 |
|  |  |  | Barts + ALL-IDB2 | 549 | 0.583 | N/A | 0.583 | 0 |
|  | SGD |  | ALL-IDB2 | 260 | 0.793 | 0.718 | 0.5 | 0.323 |
|  |  |  | Barts | 289 | 0.656 | 0 | 0.657 | 0.005 |
|  |  |  | Barts + ALL-IDB2 | 549 | 0.694 | 0.713 | 0.583 | 0.134 |
|  | Adam | ALL-IDB2 | ALL-IDB1 | 108 | N/A | 0.546 | 0.454 | 1 |
|  |  |  | Barts | 289 | N/A | 0.343 | 0.657 | 1 |
|  |  |  | Barts + ALL-IDB1 | 397 | N/A | 0.398 | 0.602 | 1 |
|  | SGD |  | ALL-IDB1 | 108 | 1 | 0.621 | 0.454 | 0.735 |
|  |  |  | Barts | 289 | 0.671 | 0.404 | 0.657 | 0.163 |
|  |  |  | Barts + ALL-IDB1 | 397 | 0.688 | 0.544 | 0.602 | 0.28 |
|  | Adam | Barts | ALL-IDB1 | 108 | 0.454 | N/A | 0.454 | 0 |
|  |  |  | ALL-IDB2 | 260 | 0.5 | N/A | 0.5 | 0 |
|  | SGD |  | ALL-IDB1 | 108 | 0.454 | N/A | 0.454 | 0 |
|  |  |  | ALL-IDB2 | 260 | 0.5 | N/A | 0.5 | 0 |
|  | Adam | ALL-IDB1 + ALL-IDB2 | Barts | 289 | 0.657 | N/A | 0.657 | 0 |
|  | SGD | ALL-IDB1 + ALL-IDB2 | Barts | 289 | 0.661 | 0.667 | 0.657 | 0.005 |
| VIT3 | Adam | ALL-IDB1 | ALL-IDB2 | 260 | 0.5 | N/A | 0.5 | 0. |
|  |  |  | Barts | 289 | 0.657 | N/A | 0.657 | 0 |
|  |  |  | Barts + ALL-IDB2 | 549 | 0.583 | N/A | 0.583 | 0 |
|  | SGD |  | ALL-IDB2 | 260 | 0.798 | 0.715 | 0.5 | 0.331 |
|  |  |  | Barts | 289 | 0.661 | 0.667 | 0.657 | 0.005 |
|  |  |  | Barts + ALL-IDB2 | 549 | 0.699 | 0.714 | 0.583 | 0.138 |
|  | Adam | ALL-IDB2 | ALL-IDB1 | 108 | N/A | 0.546 | 0.454 | 1 |
|  |  |  | Barts | 289 | 0.78 | 0.363 | 0.657 | 0.832 |
|  |  |  | Barts + ALL-IDB1 | 397 | 0.78 | 0.419 | 0.602 | 0.866 |
|  | SGD |  | ALL-IDB1 | 108 | 1 | 0.621 | 0.454 | 0.735 |
|  |  |  | Barts | 289 | 0.674 | 0.42 | 0.657 | 0.153 |
|  |  |  | Barts + ALL-IDB1 | 397 | 0.69 | 0.552 | 0.602 | 0.272 |
|  | Adam | Barts | ALL-IDB1 | 108 | 0.707 | 0.84 | 0.454 | 0.163 |
|  |  |  | ALL-IDB2 | 260 | 0.585 | 0.93 | 0.5 | 0.023 |
|  | SGD |  | ALL-IDB1 | 108 | 0.454 | N/A | 0.454 | 0 |
|  |  |  | ALL-IDB2 | 260 | 0.5 | N/A | 0.5 | 0 |
|  | Adam | ALL-IDB1 + ALL-IDB2 | Barts | 289 | 0.656 | 0 | 0.657 | 0.005 |
|  | SGD | ALL-IDB1 + ALL-IDB2 | Barts | 289 | 0.661 | 0.667 | 0.657 | 0.005 |
| VIT4 | Adam | ALL-IDB1 | ALL-IDB2 | 260 | 0.5 | N/A | 0.5 | 0. |
|  |  |  | Barts | 289 | 0.675 | 0.471 | 0.657 | 0.095 |
|  |  |  | Barts + ALL-IDB2 | 549 | 0.586 | 0.471 | 0.583 | 0.056 |
|  | SGD |  | ALL-IDB2 | 260 | 0.62 | 0.9 | 0.5 | 0.046 |
|  |  |  | Barts | 289 | 0.657 | N/A | 0.657 | 0 |
|  |  |  | Barts + ALL-IDB2 | 549 | 0.642 | 0.9 | 0.583 | 0.019 |
|  | Adam | ALL-IDB2 | ALL-IDB1 | 108 | N/A | 0.546 | 0.454 | 1 |
|  |  |  | Barts | 289 | N/A | 0.343 | 0.657 | 1 |
|  |  |  | Barts + ALL-IDB1 | 397 | N/A | 0.398 | 0.602 | 1 |
|  | SGD |  | ALL-IDB1 | 108 | 1 | 0.557 | 0.454 | 0.959 |
|  |  |  | Barts | 289 | 0.761 | 0.374 | 0.657 | 0.732 |
|  |  |  | Barts + ALL-IDB1 | 397 | 0.768 | 0.433 | 0.602 | 0.778 |
|  | Adam | Barts | ALL-IDB1 | 108 | 0.767 | 0.754 | 0.454 | 0.327 |
|  |  |  | ALL-IDB2 | 260 | 0.707 | 0.77 | 0.5 | 0.2 |
|  | SGD |  | ALL-IDB1 | 108 | 0.454 | N/A | 0.454 | 0 |
|  |  |  | ALL-IDB2 | 260 | 0.5 | N/A | 0.5 | 0 |
|  | Adam | ALL-IDB1 + ALL-IDB2 | Barts | 289 | 0.659 | 0.5 | 0.657 | 0.005 |
|  | SGD | ALL-IDB1 + ALL-IDB2 | Barts | 289 | 0.657 | N/A | 0.657 | 0 |

Figure F3. **Training and validation loss over epochs for different ViT models.** The rapid convergence (top left), lowest overall loss (top right), divergence (bottom left), and reaching the maximum number of epochs (bottom right) of different models when tested externally.


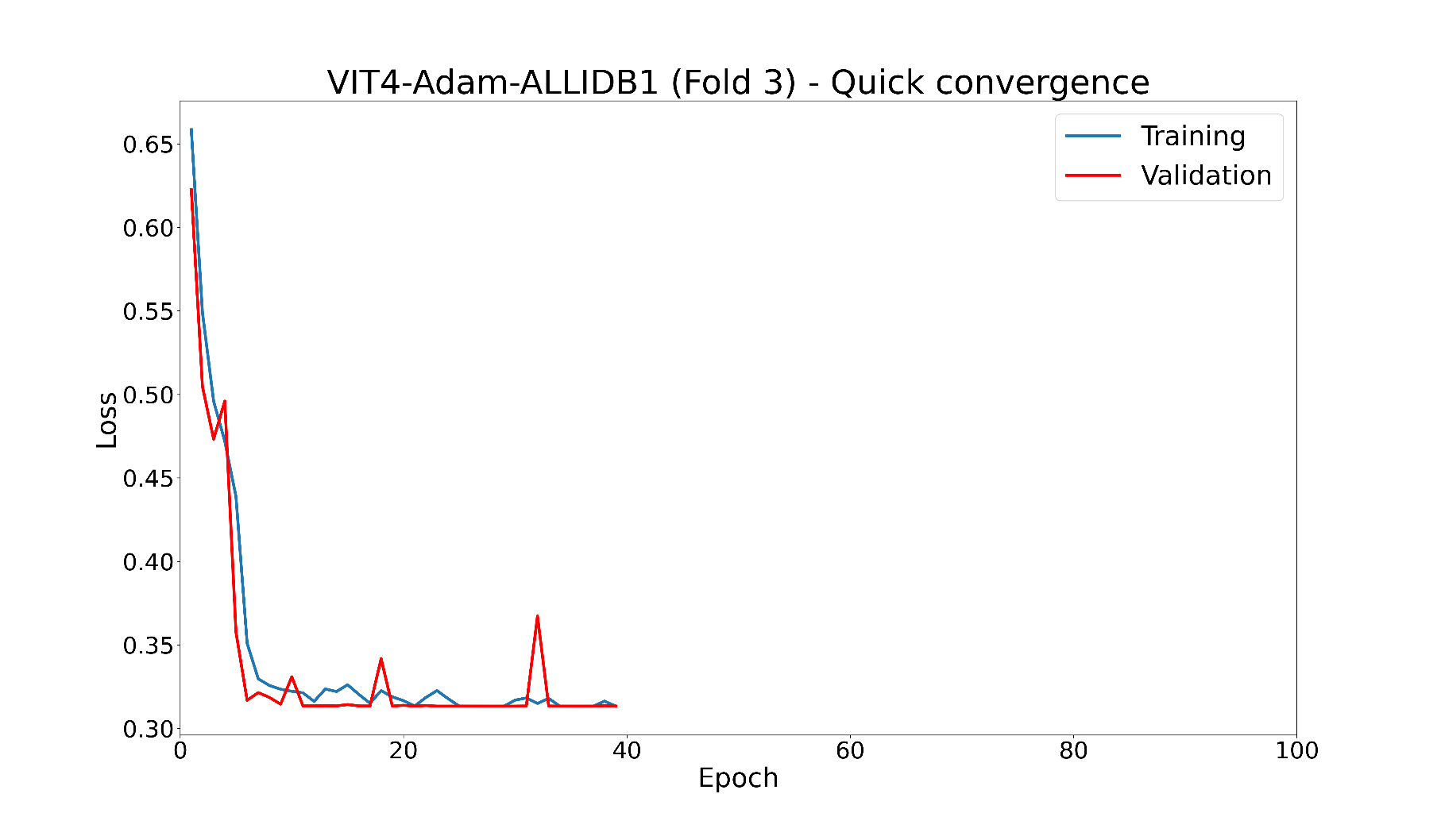

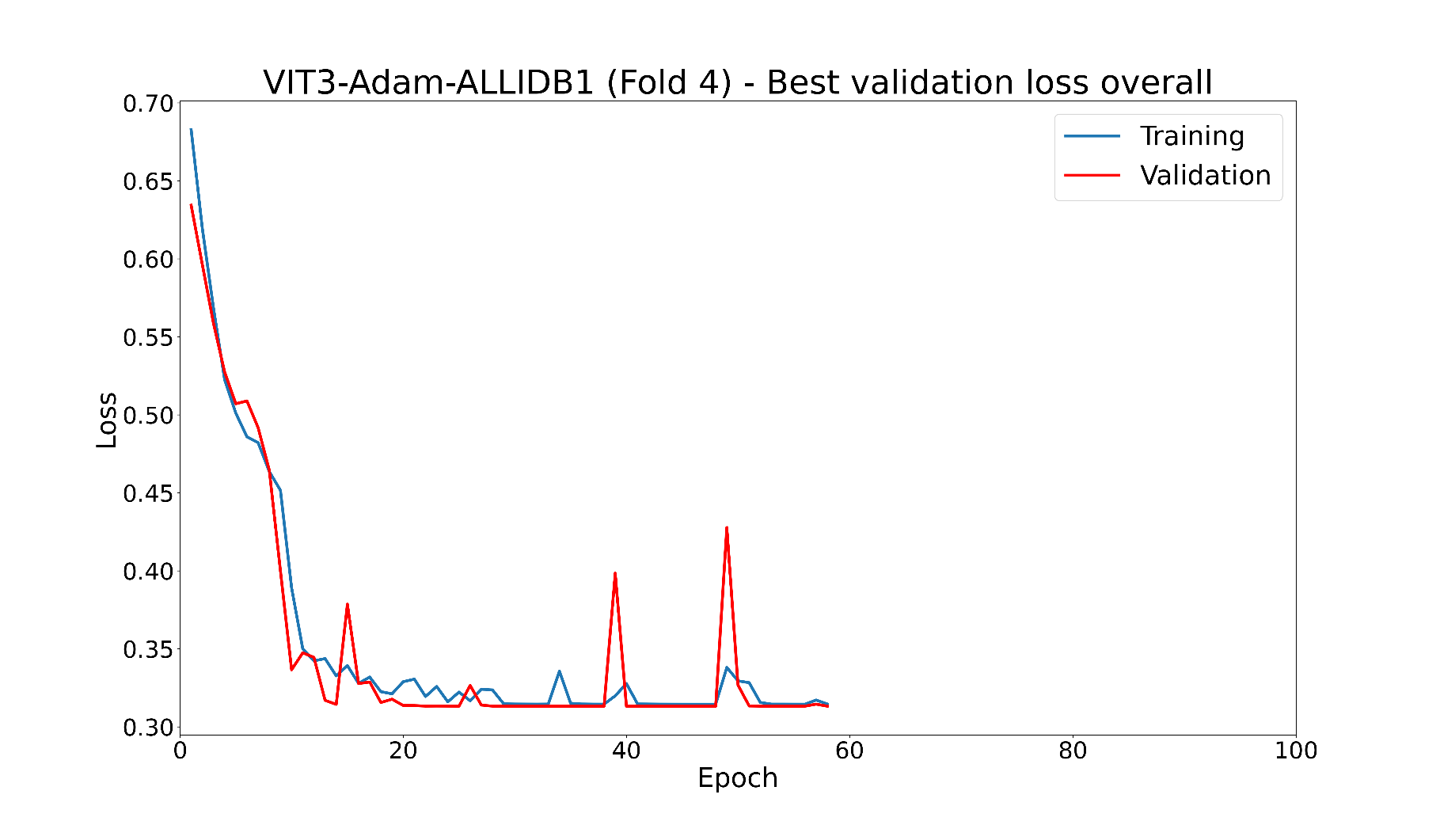


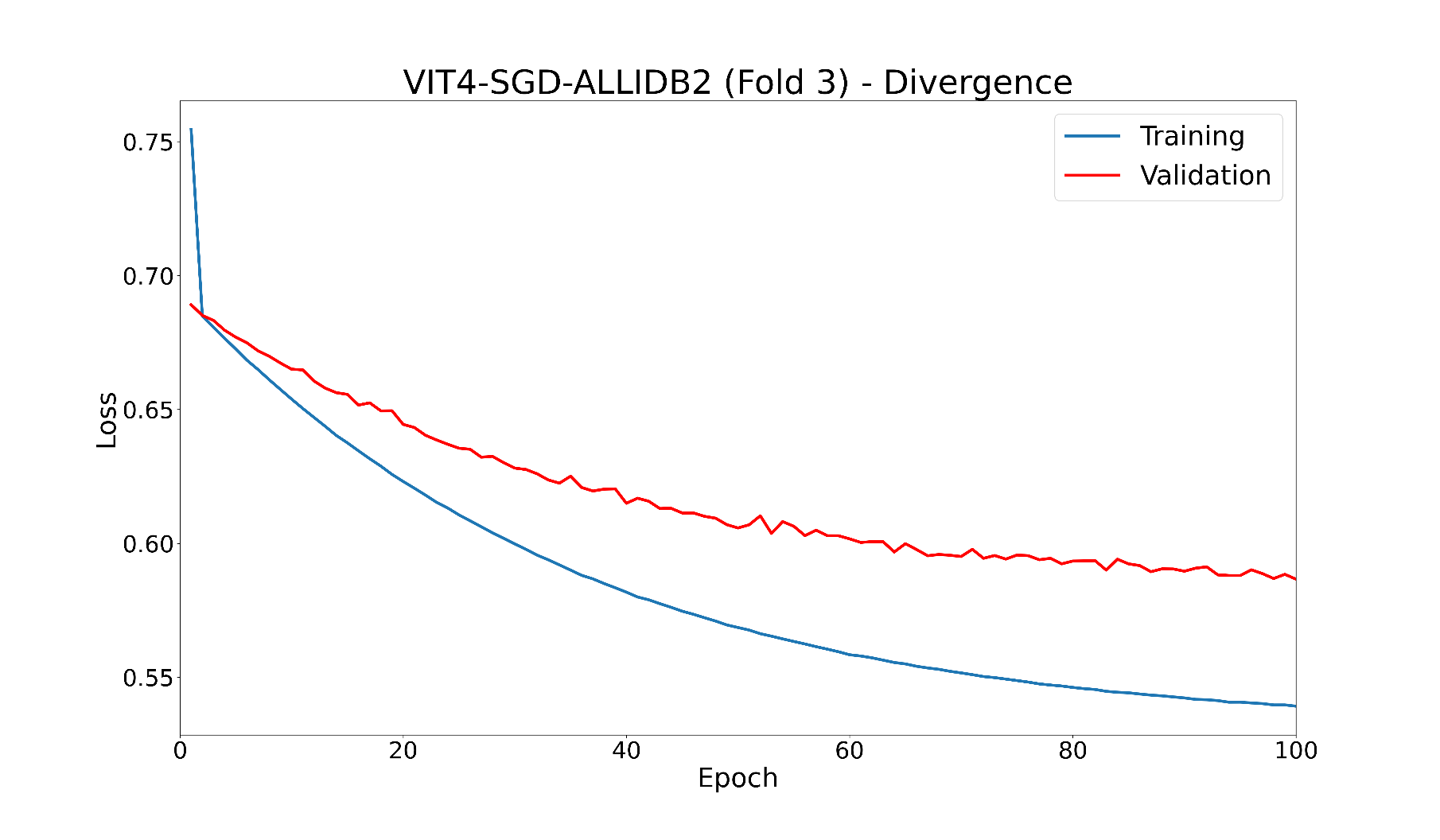

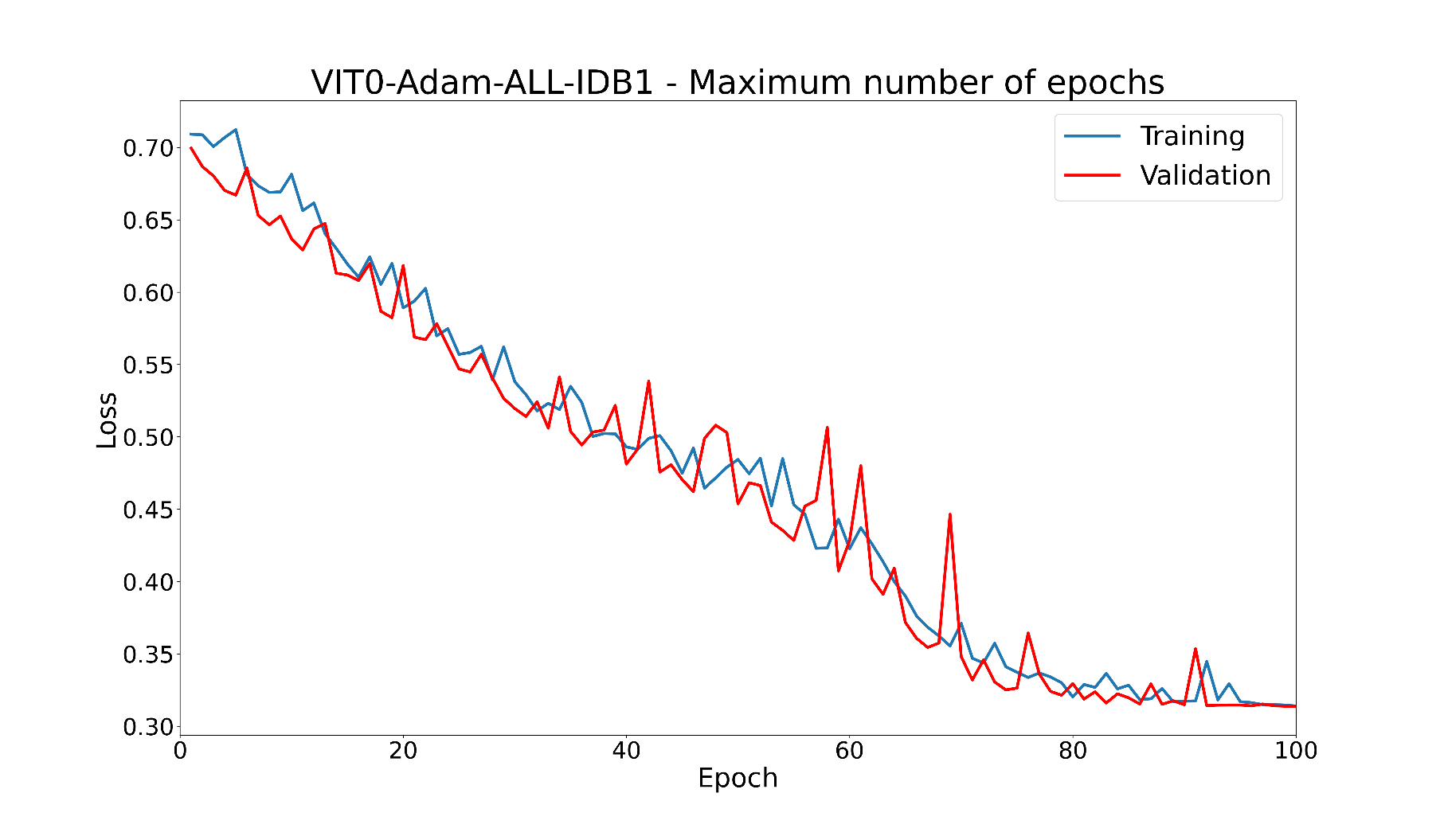

Supplement: Supplementary material — Includes Table S1, Fig F1, Table S2, Fig F2, Table S3, Table S4, and Fig F3, providing additional information to support the results referenced in the main text. [file mmc1.docx]
